# Genome-wide association study identifies new loci associated with noise-induced tinnitus in Chinese populations

**DOI:** 10.1186/s12863-021-00987-y

**Published:** 2021-09-06

**Authors:** Chengyong Xie, Yuguang Niu, Jie Ping, Yahui Wang, Chenning Yang, Yuanfeng Li, Gangqiao Zhou

**Affiliations:** 1 Medical College of Guizhou University, Guiyang City, 550025 China; 2 Department of Ambulatory Medicine, The First Medical Center of PLA General Hospital, Beijing, 100853 China; 3 State Key Laboratory of Proteomics, National Center for Protein Sciences, Beijing Institute of Radiation Medicine, Beijing, 100850 China; 4 Collaborative Innovation Center for Personalized Cancer Medicine, Center for Global Health, School of Public Health, Nanjing Medical University, Nanjing City, 210029 China

**Keywords:** Genome-wide association study, Tinnitus, Noise, *WNT11*, *TNFRSF1A*

## Abstract

**Background:**

Tinnitus is an auditory phantom sensation in the absence of an acoustic stimulus, which affects nearly 15% of the population. Excessive noise exposure is one of the main causes of tinnitus. To now, the knowledge of the genetic determinants of susceptibility to tinnitus remains limited.

**Results:**

We performed a two-stage genome-wide association study (GWAS) and identified that two single nucleotide polymorphisms (SNPs), rs2846071 located in the intergenic region at 11q13.5 (odds ratio [OR] = 2.14, 95% confidence interval [CI] = 1.96–3.40, combined *P* = 4.89 × 10^− 6^) and rs4149577 located in the intron of *TNFRSF1A* gene at 12p13.31 (OR = 2.05, 95% CI = 1.89–2.51, combined *P* = 6.88 × 10^− 6^), are significantly associated with the susceptibility to noise-induced tinnitus. Furthermore, the expression quantitative trait loci (eQTL) analyses revealed that rs2846071 is significantly correlated with the expression of *WNT11* gene, and rs4149577 with the expression of *TNFRSF1A* gene in multiple brain tissues (all *P* < 0.05). The newly identified candidate gene *WNT11* is involved in Wnt pathway, and *TNFRSF1A* in the tumor necrosis factor pathway, respectively. Pathway enrichment analyses also showed that these two pathways are closely relevant to tinnitus.

**Conclusions:**

Our findings highlight two novel loci at 11q13.5 and 12p13.31 conferring susceptibility to noise-induced tinnitus. and suggest that the *WNT11* and *TNFRSF1A* genes might be the candidate causal targets of 11q13.5 and 12p13.31 loci, respectively.

**Supplementary Information:**

The online version contains supplementary material available at 10.1186/s12863-021-00987-y.

## Background

Tinnitus is an auditory phantom sensation in the absence of an acoustic stimulus, which affects nearly 15% of the population [1]. Tinnitus can result in an impossibility to relax and depression, which may seriously reduce the life quality of the affected individuals [2]. Therefore, understanding the mechanisms of tinnitus is of great significance. However, for decades, our knowledge of phantom sounds is limited, and the occurrence, development and clinical outcome of tinnitus remain largely unknown [3].

It has been reported that excessive noise exposure was one of the main causes of tinnitus [4–6]. Additionally, ototoxic drugs, hearing loss, stress, depression, sex, drinking, smoking and history of arthritis are also relevant to the development of tinnitus [4–6]. In addition to these external risk factors, genetic factors may also be involved in tinnitus susceptibility [7]. Recently, a longitudinal male twin cohort study (*n* = 1114 at baseline and 583 at follow-up) confirmed that genetic factors do participate in the development of tinnitus [8]. Several candidate gene-based and genome-wide association studies (GWASs) have successfully identified a collection of candidate susceptibility genes for tinnitus, which can be roughly divided into the following five categories: (i) cardiovascular associated genes; (ii) neurotrophic factors associated genes; (iii) potassium recycling pathway genes; (iv) γ-aminobutyric acid type B (GABA_B_) receptor subunit associated genes; and (v) serotonin receptor/transporter associated genes [9]. Especially, a recent GWAS of noise-induced tinnitus in the Belgian population showed that several metabolic pathways are significantly associated with this disease [10]. However, no GWAS for noise-induced tinnitus in the Chinese population has been performed.

To identify novel loci related to the risk of noise-induced tinnitus in the Chinese population, we performed a GWAS; consisting of 65 noise-induced tinnitus patients (cases) and 233 subjects with normal hearing who have been exposed to a similar noise environment (controls), followed by a replication study in an independent sample set consisting of 34 cases and 379 controls. We found strong evidence for 11q13.5 and 12p13.31 as new loci contributing to susceptibility to noise-induced tinnitus. These findings expand our understanding of the genetic susceptibility to tinnitus.

## Results

### Genome-wide association analyses

**Fig. 1:**
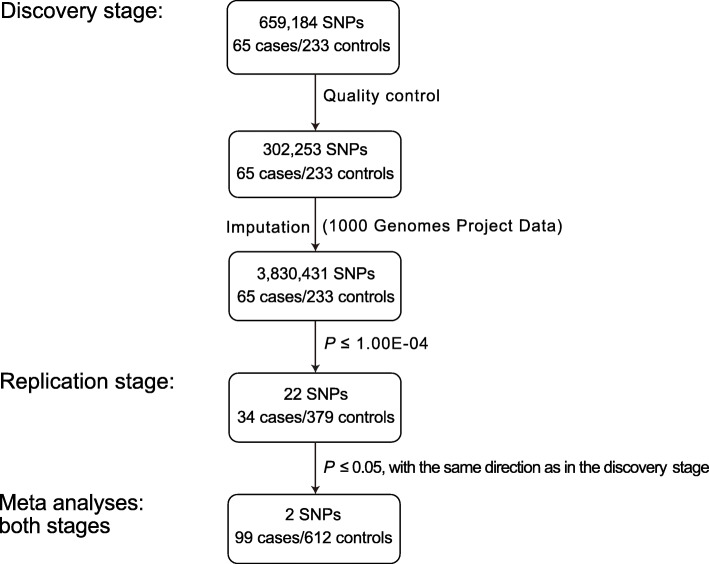
An overview of the study workflow. Numbers refer to the sample sizes of the cases and controls, and the numbers of single nucleotide polymorphisms (SNPs) that were genotyped or imputed. The imputation was performed using the data from all populations from the 1000 Genomes Project (phase 3) and generated genotypes of a total of 3,830,431 SNPs. The 22 top significantly associated SNPs in the discovery stage were genotyped in the samples of the replication stage. Two SNPs, rs2846071 and rs4149577, were replicated in the replication stage. Lastly, meta-analyses combining two stages for rs2846071 and rs4149577 were performed

To identify novel loci conferring susceptibility to noise-induced tinnitus among Chinese populations, we performed a two-stage GWAS (Fig. 1). In the discovery stage, we used the Illumina Infinium Asian Screening Array-24 (v1.0) to genotype the 659,184 single nucleotide polymorphisms (SNPs) in 65 noise-induced tinnitus patients (cases) and 233 non-tinnitus individuals (controls) (Table 1 and Table S1). After quality controls, a total of 302,253 autosomal SNPs in these 298 individuals were retained, with an average genotyping call rate > 99.8% (Table S2). No outlier was presented using the principal component analysis (PCA) (Fig. S1a). PCA also showed that the cases and controls are genetically well-matched, and all these subjects are of Chinese ancestry (Fig. S1b). No significant principal components (PCs) were found using the Tracy-Widom statistic.

**Table 1:** Summary of the case/control populations used in the discovery and replication stages

Stages	Cases		Controls	
	Sample size	Mean age (s.d.)	Sample size	Mean age (s.d.)
Discovery stage	65	23.8 (1.6)	233	23.4 (1.6)
Replication stage	34	26.4 (3.9)	379	24.5 (2.9)
Overall	99	24.7 (2.9)	612	24.1 (2.5)

*GWAS* genome-wide association study, *s.d* standard deviation

**Fig. 2:**
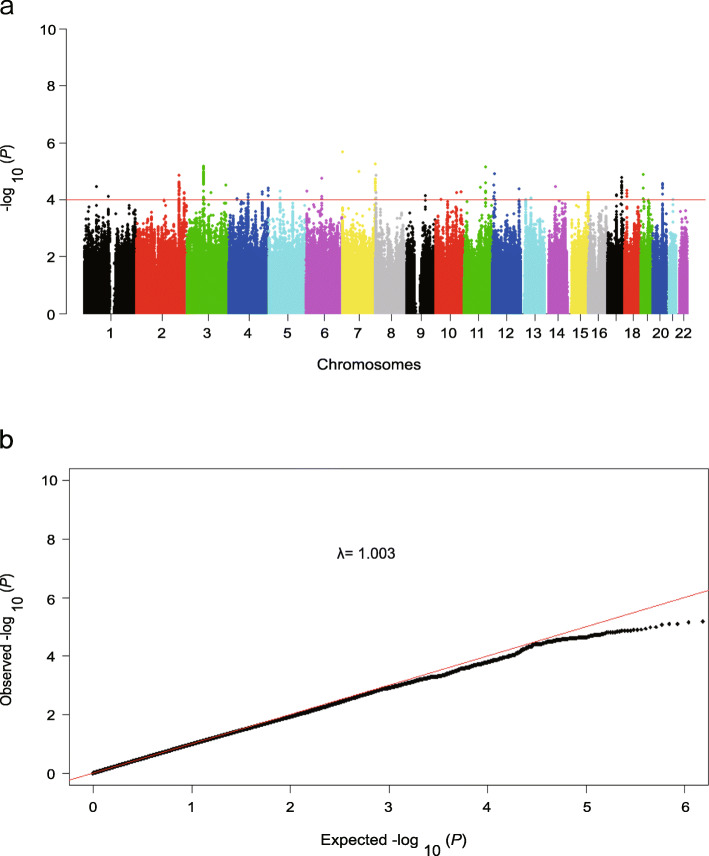
Manhattan plot and Quantile-quantile plot of the genome-wide *P* values from the association test on tinnitus. **a** The Manhattan plot of genome-wide *P* values for the genotyped and imputed SNPs using logistic regression analyses in the cases/controls population in the discovery stage under the additive model. The x-axis represents the genomic position (based on NCBI Build 37), and the y-axis shows the -log10 (*P*). **b** The quantile-quantile plot. The red line represents the null hypothesis of no true association. The black line with gradient λ (inflation coefficient) is fitted to the lower 90% of the distribution of the observed test statistics. The plot is based on the genotyped and imputed SNPs that passed the quality controls. The value of the inflation factor λ is 1.003 under the additive model

After imputation in strict accordance with the standard process, we achieved a total of 3,830,431 SNPs (Table S2). We then carried out genotype-phenotype association analyses by the logistic regression model, with adjustment for age and noise exposure time. A manhattan plot showed the associations between the genome-wide SNPs and the risk of tinnitus (Fig. 2a). However, none of the SNPs reached the threshold for genome-wide significance (*P* < 5.0 × 10^− 8^). A quantile-quantile (Q-Q) plot was performed and showed a good match between the distributions of the observed *P* values and those expected by chance (inflation factor *λ* = 1.003; Fig. 2b), suggesting minimal overall inflation of the genome-wide statistical results in the discovery stage.

Several candidate gene-based association studies and a GWAS for tinnitus have identified several SNPs that were significantly associated with the risk of tinnitus [9]. In the present study, however, these SNPs did not show any significant association with noise-induced tinnitus (Table S3). These results are unlikely to be genotyping or imputation errors, since these SNPs were genotyped or imputed with high quality. The inconsistent associations may be due to the following reasons: (1) Population heterogeneity. The previously reported significantly associated SNPs in *KCNE1* (index SNP rs915539) and *KCTD12* (index SNP rs34544607) were monomorphic in the Chinese population (Table S3); (2) Limited sample sizes. The sample sizes in this study and most of the previous studies are less than 1000; (3) Different experimental designs. The experimental design in this study is to identify susceptibility genes in noise-induced tinnitus, while many previous studies aimed to identify susceptibility genes in other types of tinnitus, such as drug-induced or age-induced tinnitus; and (4) Different sample collecting methods. The controls in this study are the non-tinnitus subjects exposing to the noise, while the controls in many previous studies are naïve controls.

### Two new susceptibility SNPs at 11q13.5 and 12p13.31 were identified

**Fig. 3:**
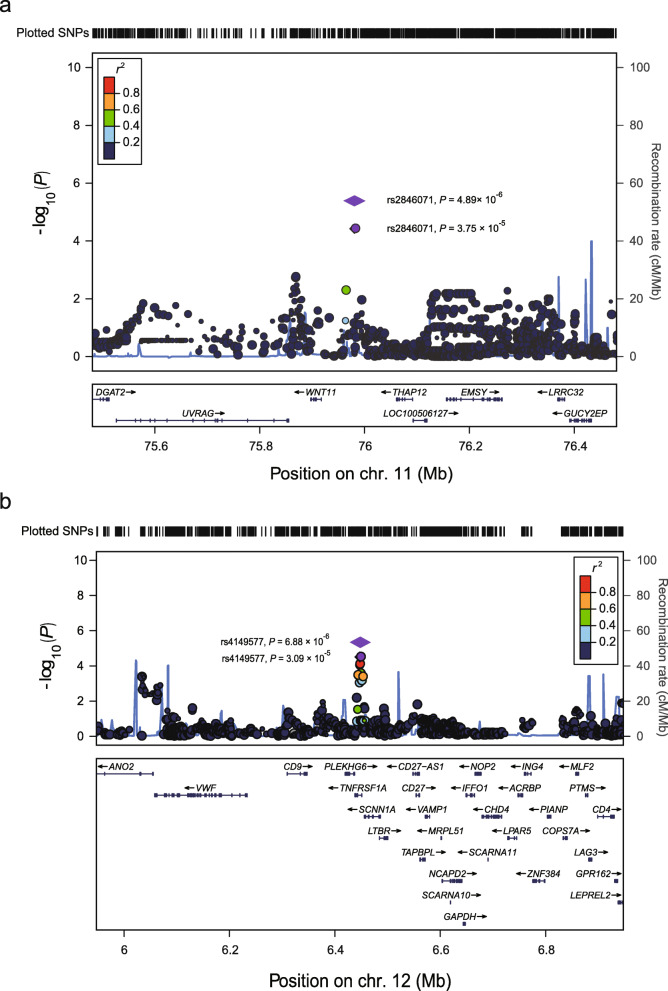
Regional plots for the associations in regions surrounding the rs2846071 or rs4149577 in the discovery stage. Genomic positions are based on NCBI Build 37. In the meta-analysis, the *P* value of the SNP is shown as purple diamonds, with their initial *P* value in the discovery stage shown as purple dots. The linkage disequilibrium (LD) values (*r*^2^) to rs2846071 or rs4149577 for the other SNPs are indicated by marked color. Red signifies *r*^2^ > 0.8, orange 0.6 < *r*^2^ ≤ 0.8, green 0.4 < *r*^2^ ≤ 0.6, light blue 0.2 < *r*^2^ ≤ 0.4 and blue *r*^2^ ≤ 0.2. Estimated recombination rates, which are derived from the East Asian populations of the 1000 Genomes Project (phase 3), are plotted in blue. Genes within the 500 kb region surrounding the index SNPs rs2846071 (**a**) or rs4149577 (**b**) are annotated, with the positions of transcripts shown by arrows. The East Asian populations from the 1000 Genomes Project consist of 504 subjects from the CHB (Han Chinese in Beijing, China), CHS (Southern Han Chinese), CDX (Chinese Dai in Xishuangbanna, China), JPT (Japanese in Tokyo, Japan) and KHV (Kinh in Ho Chi Minh City, Vietnam)

We selected 22 index SNPs at 22 loci for replication in an independent population, which consists of 34 cases and 379 controls (Tables S4 and S5). Among these 22 index SNPs, two SNPs showed significant associations with the risk of tinnitus in the same direction as those observed in the discovery stage (*P* = 0.024 for rs2846071 and *P* = 0.049 for rs4149577, respectively; Table S6). In addition, we conducted meta-analyses for these two SNPs based on the results of the discovery and replication stages. Both SNPs showed more significant associations (odds ratio [OR] = 2.14, 95% confidence interval [CI] = 1.96–3.40, *P* = 4.89 × 10^− 6^ for rs2846071; and OR = 2.05, 95% CI = 1.89–2.51, *P* = 6.88 × 10^− 6^ for rs4149577; Table 2 and Fig. 3). No evidence for heterogeneity of OR values for rs2846071 and rs4149577 was observed across the populations from the discovery and replication stages (*P*_heterogeneity_ = 0.27 and 0.31, respectively; Table 2).

**Table 2:** Association results for the rs2846071 and rs4149577 in the case/control populations

SNPs	Chr. (Cytoband)	Studies	Cases^a^	Controls^a^	ORs (95% CIs)	*P* values	I^2^	*P*_heterogeneity_
rs2846071	11q13.5	Discovery stage	14/36/15	16/107/110	2.54 (1.63–3.96)	3.75 × 10^−5^	19.07	0.27
T/C^b^		Replication stage	11/12/11	50/161/157	1.75 (1.08–2.84)	0.024		
		Overall	25/48/26	66/277/267	2.14 (1.96–3.40)	4.89 × 10^−6^		
rs4149577	12p13.31	Discovery stage	19/31/15	26/93/114	2.33 (1.56–3.46)	3.09 × 10^−5^	1.28	0.31
A/G^b^		Replication stage	8/16/10	43/178/155	1.67 (1.00–2.78)	0.049		
		Overall	27/47/25	69/271/269	2.05 (1.89–2.51)	6.88 × 10^−6^		

*Chr.* chromosome, *CI* confidence interval, *OR* odds ratio, *SNP* single nucleotide polymorphism. ^a^Counts of TT/TC/CC genotypes for rs2846071 and AA/AG/GG genotypes for rs4149577 in the case/control populations, respectively. These two SNPs were genotyped using the Illumina Infinium Asian Screening Array-24 (v1.0) in the discovery stage. The number of genotyped samples varies due to genotyping failure. ^b^Minor allele/major allele. ORs and 95% CIs were calculated under the additive model by logistic regression while adjusting for the age and noise exposure time

We further investigated whether the age of subjects has a modification effect on the association between these two SNPs (rs2846071 and rs4149577) and tinnitus. We found no significant changes in the effects of rs2846071 and rs4149577 on the risk of noise-induced tinnitus when stratified by age (*P*_heterogeneity_ = 0.80 and 0.52, respectively; Table S7). Since the participants in the discovery and replication stages of this study are all males, so the interference of sex-related factors could be ruled out.

### Chromosome 11q13.5 locus

The index rs2846071 was located in the intergenic region at chromosome 11q13.5. Seven protein-coding genes (*DGAT2*, *UVRAG*, *WNT11*, *THAP12*, *EMSY*, *LRRC32* and *GUCY2EP*) are located within the 500 kb region surrounding this SNP (Fig. 3a). We performed the expression quantitative trait loci (eQTL) analyses based on the datasets of brain tissues from the Genotype-Tissue Expression (GTEx, release v8) to identify the candidate causative genes at 11q13.5. We used eQTL data of the brain tissues, because several pieces of evidence have supported the relevance of brain tissues to tinnitus. For examples, several neuroscience studies have demonstrated that most tinnitus cases developed tinnitus as a consequence of changes that occur in central auditory pathways and other brain regions [11, 12]. Moreover, the mice model studies have confirmed that tinnitus-related changes started in the cochlear nucleus and extended to the auditory cortex and other brain regions [11, 12]. The eQTL analysis showed that the index rs2846071 is significantly associated with the expression levels of *WNT11* in the brain anterior cingulate cortex, cerebellum and cortex (*P* = 0.047, 7.7 × 10^− 4^ and 1.5 × 10^− 4^, respectively; Fig. S2), but not with the expressions of other genes. We further performed colocalization analyses for GWAS and eQTL signals using the R package “Coloc” (3.2.1). However, the colocalization analyses showed that the posterior probability of hypothesis 4 (PP4) of rs2846071-*WNT11* is less than 0.2 (PP4 = 0.025), indicating that the tinnitus-associated SNP rs2846071 is not colocalized with eQTL signal for *WNT11* in brain tissues (Figs. S3a-c). We also found that no nearby genes pass the PP4 threshold of 20% (data not shown). Indeed, *WNT11* was identified as the top gene in the colocalization analyses. Taking this together with the significant eQTL results for *WNT11* in the brain tissues, we suggested that *WNT11* may be the candidate gene at this locus. Further studies are needed to confirm this hypothesis.

The causal SNP is not necessary the most statistically significant SNP [13]. Given this, we performed a functional annotation on the genetic variants tagged by index SNP rs2846071 to investigate the candidate causative variants at 11q13.5. Based on Haploreg (v4.1), eight SNPs at 11q13.5 are shown to be in strong or moderate linkage disequilibrium (LD) with rs2846071 (1 ≥ *r*^2^ ≥ 0.4), spanning ~ 15 kb genomic regions (Table S8a). Using the PAINTOR software, we obtained the posterior probabilities for these 8 SNPs. Among these 8 SNPs, the rs2846071 has the highest posterior probability (0.48; Table S8a), suggesting that this SNP may be the causal SNP in this locus. Roadmap Epigenomics Consortium data revealed that rs2846071 is located in the enhancer region in multiple human brain tissues (Table S8a). Together, we thus speculated that rs2846071, or another in LD, may be the variant that has a causal effect on the risk of tinnitus by regulating *WNT11* gene expression in brain tissues.

### Chromosome 12p13.31 locus

The index SNPs rs4149577 was located at chromosome 12p13.31. More than 10 protein-coding genes were located in the 500 kb region surrounding rs4149577 (Fig. 3b). We performed eQTL analyses based on the 13 types of brain tissue in GTEx to identify the potentially causative gene(s) at 12p13.31. The eQTL analyses showed that the genotypes of rs4149577 are significantly associated with the expression levels of tumor necrosis factor receptor superfamily member 1A (*TNFRSF1A*) in the brain caudate, cerebellar hemisphere, cerebellum, cortex, frontal cortex and putamen tissues (all *P* < 0.05; Fig. S4). Colocalization analysis further showed that the tinnitus-associated SNP rs4149577 is colocalized with eQTL signals for *TNFRSF1A* in brain tissues (PP0 = 0.040, PP1 = 0.002, PP2 = 0.613, PP3 = 0.057, PP4 = 0.288; Figs. S3d-f) [14]. Together, these pieces of evidence suggested a potential role for *TNFRSF1A* in the development of tinnitus.

To identify the potential causal variants at the 12p13.31 locus, we performed a functional annotation on the 8 SNPs tagged by index SNP rs4149577 (*r*^2^ > 0.4), which span ~ 14 kb region (Table S8b). By using the PAINTOR software, we got the posterior probability of these 8 SNPs at 12p13.31. Among them, rs1800692 and rs4149570 had the highest posterior probability (1.00; Table S8b). We performed eQTL analyses for these two SNPs and showed that the genotypes of these two SNPs are significantly associated with *TNFRSF1A* in multiple brain tissues (all *P* < 0.05). The most significant eQTL results for these two SNPs were in the brain caudate tissues (*P* = 1.9 × 10^− 5^ and 4.8 × 10^− 5^, respectively). Further, these two SNPs were predicted to be located in enhancer and promoter signals in human brain tissues based on Roadmap Epigenomics Consortium data (Table S8b). Additionally, chromatin state segmentation by hidden markov model (HMM) from ENCODE/Broad database showed that these two SNPs are located in enhancer regions in various types of cells (Fig. S5). Together, these results suggested that these two SNPs may be the candidate causative variants in this region.

### Pathway enrichment analyses

To investigate the pathways or biological processes potentially involved in noise-induced tinnitus, we employed the i-GSEA4GWAS, which is a tool using the summary statistics of all SNPs from the GWAS, without restricting the analyses to a significance threshold [15]. In total, five pathways showed significant associations with noise-induced tinnitus, including the arachidonic acid metabolism, inositol phosphate metabolism, Notch signaling, Wnt signaling and tumor necrosis factor (TNF) pathways (all *P* < 0.05; Table S9). Among these pathways, the arachidonic acid metabolism and inositol phosphate metabolism showed the strongest association (*P* < 0.001; Table S9). This result was consistent with previous GWAS findings that the metabolic pathways were significantly associated with tinnitus [10]. It has been reported that the altered arachidonic acid (a substrate of cyclooxygenase) metabolism may be the physiological basis of salicylate-induced tinnitus [16]. The inositol phosphate has been shown to induce Ca^2+^ elevation in cochlear sensory epithelial cells [17]. Additionally, several other pathways may be involved in tinnitus. For example, Wnt signaling and Notch signaling could regulate each other [18, 19], and are required for supporting cell proliferation and hair cell differentiation in the cochlea [20]. TNF pathway could induce apoptosis of auditory hair cells in vitro in hair cell nuclei [21]. Intriguingly, the newly identified significantly associated genes *WNT11* and *TNFRSF1A* were just in the Wnt and TNF pathways, respectively, therefore highlight the critical roles of these two pathways in the development of tinnitus.

## Discussion

In the present study, we performed a GWAS of noise-induced tinnitus in the Chinese population. To our best knowledge, this is the first GWAS for the risk of tinnitus among Chinese population. We successfully identified two novel loci at 11q13.5 (index rs2846071) and 12p13.31 (index rs4149577) loci, which were significantly associated with the susceptibility to noise-induced tinnitus.

We compared the allele frequencies of these two SNPs with those in the main populations from the 1000 Genomes Project (phase 3). On the one hand, we found that the allele frequency of rs2846071 [T] (0.356) is similar to that of East Asian descent (0.354, *P* = 0.94) in the 1000 Genomes Project, but significantly lower than that of Europeans, Africans and Americans descent (*P* = 1.56 × 10^− 53^, 2.39 × 10^− 110^ and 1.97 × 10^− 15^, respectively; Table S10). On the other hand, we found that the SNP rs4149577 [A] allele frequency (0.360) is similar to that of the East Asian descent (0.360, *P* = 0.77), but significantly lower than that of the Europeans, Africans and Americans descent (*P* = 2.15 × 10^− 16^, 2.25 × 10^− 204^ and 1.64 × 10^− 8^, respectively; Table S10). Further studies are needed to investigate whether the difference in allele frequencies of these SNPs among different ethnic groups affects the susceptibility to tinnitus.

The two identified SNPs in this study are all located in non-coding regions, which may affect the disease risk by regulating the transcription levels of related genes [22]. The eQTL analysis is helpful to reveal the relationship between the genetic variation and expression of the nearby genes in specific tissue types [23]. Here, our eQTL analyses showed that the genotypes of rs2846071 or rs4149577 are correlated with the expression levels of *WNT11* or *TNFRSF1A*, respectively, in multiple brain tissues, suggesting that these two genes may be the candidate genes of tinnitus. However, the analyses of eQTL were complicated due to tissue heterogeneity. In addition, most of the samples in the GTEx database are of European ancestry, while the samples in this GWAS were all of Chinese ancestry. The differences in LD values and allele frequencies between the Chinese population and the European population will potentially influence the eQTL and colocalization signals (Figs. S6 and S7). Thus, it is necessary to be cautious when interpreting the results of the eQTL and colocalization analyses. Further analysis was needed in a larger sample size study to confirm the genetic associations of *WNT11* and *TNFRSF1A* with tinnitus.

The *WNT11* gene encodes a secretory signal protein, which is involved in the Wnt pathway [24]. WNT11 has been reported to involve the formation of cilia [25], and the abnormality of cilia could influence tinnitus occurrence [26]. Besides, the Wnt pathway was considered to be the key to many basic development processes, including the hearing items [27]. For example, knock-out of β-catenin in mice has been shown to inhibit the differentiation of hair cells as well as columnar cells from sensory progenitor cells [28]. Besides, Wnt activation could protect against hair cell damage in the mouse cochlea [29]. Mutations in several genes of the Wnt/planar cell polarity (PCP) pathway (such as *Wnt11* and *Gpc4*) could result in the misorientation of hair cells in mice [30]. *TNFRSF1A* encodes a member of TNF receptor superfamily of proteins and was involved in TNF pathway [31]. TNFRSF1A has been shown to involve in the production of ototoxic reactive oxygen species and has been demonstrated to be specifically upregulated in gentamicin-mediated ototoxicity [32], suggesting that high expression of *TNFRSF1A* may have damaging effects on hearing and hair cells [32]. Besides, TNFRSF1A may cause infiltration of inflammatory cells, which is known to be the main cause of hearing problems [33]. As for the TNF pathway, genetic knockout of tumor necrosis factor-alpha (TNF-α) or pharmacologically blocking TNF-α expression ameliorated the behavioral phenotype associated with noise-induced tinnitus in mice [34]. Together, these pieces of evidence suggested potential roles for WNT11 and TNFRSF1A in the development of tinnitus.

We also performed meta-analyses for all of the 22 candidate SNPs in the discovery and replication stages. We found that in addition to the reported two SNPs rs2846071 and rs4149577, another SNP rs10771523 also shows a more significant association in the meta-analysis (*P* = 1.70 × 10^− 6^) than that in the discovery stage. However, the association between rs10771523 and tinnitus in the replication stage was not significant (*P* = 0.26). Further genetic association studies are needed to replicate whether rs10771523 is significantly associated with tinnitus.

In the pathway analyses, we noted that the top two enriched pathways are metabolism pathways: arachidonic acid metabolism and inositol phosphate metabolism. These findings are consistent with a previous GWAS, which also suggested that several metabolic pathways (such as serotonin reception mediated signaling) are significantly associated with tinnitus [10, 35]. Indeed, tinnitus is considered to be a result of metabolic, neurologic and psychogenic disorders [36]. Thus, our results further highlighted the important roles of metabolism pathways in the development of tinnitus.

Up to now, two GWASs of tinnitus in European populations have been reported [10, 37]. The first GWAS of tinnitus consists of 167 tinnitus subjects and 749 non-tinnitus subjects [10], and none of the SNPs reach the threshold for genome-wide significance (*P* < 5.0 × 10^− 8^). However, several metabolic pathways showed significant associations. The other GWAS of tinnitus consists of 14,829 tinnitus subjects 119,600 subjects who have never experienced tinnitus [37]. One SNP (rs4906228) upstream of the *RCOR1* gene showed genome-wide significant association with tinnitus (*P* = 1.7 × 10^8^). Together, these results suggested that GWAS can identify interesting candidate genes for tinnitus, and these candidate genes deserve further investigation.

The advantage of this study is that the selection of the case-control population is strict, with the cases and controls exposing to the same intensity and time of noise exposure in the same environmental conditions. All the subjects were male, therefore excluding the potential confounding caused by sex. However, this study also has its shortcomings. For example, the sample size in the initial GWAS discovery stage in this study is not sufficient enough to determine all possible genetic susceptibility loci associated with noise-induced tinnitus. Therefore, the potential SNPs associated with the disease may be missed in the present study. Due to the same reason, no SNP in our results reached genome-wide significance, our conclusion is therefore reasonable speculation [38].

However, our GWAS reveals enough statistical power in 65 cases and 233 controls to detect the index rs2846071 (OR = 2.54; minor allele frequency [MAF] = 0.341) and rs10081191 (OR = 2.33; MAF = 0.359), with the estimated powers to be ~ 94% and ~ 85%, respectively (Fig. S8). Besides, the rare variation is difficult to be discovered by GWAS technology, which may also lead to the “missing heritability” of tinnitus.

## Conclusions

In summary, we conducted the first GWAS of noise-induced tinnitus in Chinese populations and identified novel loci at 11q13.5 and 12p13.31. We suggested that *WNT11* and *TNFRSF1A* may be the susceptible genes at 11q13.5 and 12p13.31, respectively. Further functional studies are warranted to establish the roles of these two loci in the pathogenesis of tinnitus. These findings advanced our understanding of the genetic mechanism of noise-induced tinnitus, and might be helpful in identifying the high-risk groups of tinnitus among noise workers, and in improving the treatment of this disease.

## Methods

### Study participants

In the present study, we performed a two-stage GWAS, totally consisting of 711 male subjects of Chinese ancestry. The discovery stage contains 298 subjects who were recruited from occupational noise-exposed workers from a single factory in March, 2018 from Bengbu city in Anhui province, China (Table S1). The replication stage contains 413 subjects, who were recruited from the same factory between August, 2018 and September, 2019 (Table S1).

These workers were exposed to noise greater than 100 dB (dB) for more than 8 h per day for at least half a year. All the subjects underwent pure tone audiometry by qualified audiologists in a standard soundproof room using a Madsen Voyager 522 audiometer (Kastrup, Denmark) according to the standard procedures. Air conduction hearing thresholds were measured for tonal stimuli at the frequencies of 0.25, 0.5, 1, 2, 4 and 8 kHz (kHz). In addition, all the subjects completed a detailed questionnaire on tinnitus, medical history and exposure to environmental risk factors, including demographic factors, noise exposure time, noise exposure intensity, a hearing threshold of both ears after noise exposure. Subjects with hearing-related complications, ear trauma, certain drugs or toxins, and otitis media were excluded.

The noise-induced tinnitus patients (cases) were defined according to the 2009 Guidelines for diagnosis and Treatment of Tinnitus (Proposal) [39]. Briefly, the subjects with persistent tinnitus after noise exposure, or with non-persistent tinnitus after noise exposure (more than 3 times) were defined as the cases. The persistent tinnitus was tinnitus lasting more than 6 months, which often negatively affects the patient’s quality of life [39]. All the noise-induced tinnitus patients completed a detailed Tinnitus Handicap Inventory (THI) questionnaire in this study to evaluate the severity of tinnitus [40]. Subjects without tinnitus were defined as controls. According to these criteria, the discovery stage contains 65 cases and 233 controls, and the mean age of the cases (23.8) is slightly higher than that of controls (23.4) (*P* = 0.080; Table S1). The replication stage contains 34 cases and 379 controls, and the mean age of cases (26.4) is significantly higher than that of controls (24.5) (*P* = 0.0070; Table S1). All the cases and controls are males, therefore excluding the potential confounding caused by sex.

### Genotyping and quality controls in the discovery stage

In the discovery stage, we genotyped all the participants using the Illumina Infinium Asian Screening Array-24 (v1.0). We performed strict quality controls for samples and SNPs to ensure the subsequent robust association tests [41]. Briefly, we removed the samples that: (i) had overall call rates of < 90%; (ii) showed sex ambiguous; or (iii) were identified as outliers by the PCA. Genome-wide Complex Trait Analysis software (GCTA; v1.92.2) was used for PCA to detect the outliers [42]. SNPs were retained if they had: (i) a call rate of > 90%; (ii) a MAF of > 0.05; (iii) did not map to the sex chromosomes; and (iv) *P* values of great than 1.0 × 10^− 4^ in a Hardy-Weinberg equilibrium test. Finally, a total of 302,253 SNPs, and 65 cases and 233 controls remained for subsequent analyses (Tables S1 and S2).

### SNP imputation

To increase the coverage of SNPs and get more genotypes in the discovery stage, we performed imputation based on the GWAS genotyping data in the discovery stage using the SHAPEIT (v2) [43] and IMPUTE2 (v2.3.1) [44] software. The 1000 Genomes Project data (phase 3) from all populations was used as the reference dataset. The posterior probability of 0.90 was used as the threshold of genotyping. The imputed probabilities were then converted to hard genotype calls. For the imputed SNPs, we also performed the quality controls to screen well-qualified SNPs. SNPs were retained if they had: (i) IMPUTE2 info > 0.6; (ii) a call rate of > 90%; (iii) a MAF of > 0.05; and (iv) a *P* value of great than 1.0 × 10^− 4^ in a Hardy-Weinberg equilibrium test. Finally, a total of 3,830,431 SNPs was obtained after strict quality controls among 65 cases and 233 controls in the discovery stage (Table S2). We also used the Michigan Imputation Server the includes 1654 individuals from the GenomeAsia 100 K Project to improve imputation since this reference panel is restricted to Asians. We, however, achieved similar results [45].

### Genome-wide association analyses in the discovery stage

Association analyses between each SNP and the risk of noise-induced tinnitus were performed using PLINK (v1.9) [46], which used logistic regression analyses under an additive model with adjustment for age and noise exposure time. Because we did not find any significant principal components (PCs) from the Tracy-Widom statistic, we didn’t adjust for PCs in the logistic regression model. The Manhattan plot of -log10 (*P* values) was generated to show the associations between the SNPs and the risk of tinnitus. The quantile-quantile plot was generated to assess the potential impact of population stratification and evaluate the overall significance of the genome-wide associations. The Manhattan and quantile-quantile plots were created with package qqman (version 0.1.8) in R (version 4.0.3). A lambda (*λ*) inflation factor is given to indicate whether the systematic bias is present.

### SNPs selection and genotyping in the replication stage

A total of 232 SNPs with *P* values ≤1.0 × 10^− 4^ in the discovery stage were chosen for the replication study. To select the SNPs to enter into the replication stage, we employed the tagger algorithm implemented in the Haploview (version 4.2) software to select the tag-SNPs among 232 SNPs in the study [47]. We first screened out the SNPs with *r*^2^ ≥ 0.8 and MAF ≥ 0.05 in the haplotype block with Haploview software, and then selected the SNPs with the mean maximum *r*^2^ as the tag-SNPs in the haplotype block [48, 49]. Thus, we achieved a total of 22 loci among these 232 SNPs. Then, these 22 tag-SNPs with significant *P* values in each locus (*P* values ≤1.0 × 10^− 4^), which were designated as the index SNPs, were selected for genotyping in the subsequent replication stage. These 22 index SNPs were genotyped using the Sequenom assays. First, locus-specific PCR and primers were designed for the 22 index SNPs using the MassARRAY Assay Design 3.0 software (Sequenom, Inc. USA). Then, approximately 15 ng of the genomic DNA for each sample was used to genotype these SNPs. The DNA samples were amplified by multiplex PCR, and the products were then used for locus-specific single-base extension reactions. The resulting products were desalted and transferred to a 384-element SpectroCHIP array (Sequenom, Inc. USA). Allele detection was performed using MALDI-TOF-MS (Sequenom, Inc. USA). The mass spectrograms were analyzed by the MassARRAY TYPER software. The cluster patterns of the genotyping data from the Sequenom assays were visually checked to confirm their good quality. Lastly, the genotype data in the replication stage was subjected to the same quality control analyses as in the discovery stage. Among the 22 SNPs, rs148091530 was failed to be genotyped. Association analyses between each SNP and the risk of noise-induced tinnitus were performed using PLINK (v1.9), which used logistic regression analyses under an additive model with adjustment for age and noise exposure time. Finally, only the rs2846071 and rs4149577 were survived in the replication stage (*P* < 0.05 and with effects in the same direction as that in the discovery stage).

### Genotype-expression association analyses

Several neuroscience studies have found that the nerve changes related to tinnitus start in the cochlear nucleus and extend to the auditory cortex and other brain regions [11]; therefore, we evaluated the genotype-specific expressions for rs2846071 and rs4149577 in 13 types of human brain tissue using the eQTL analyses based on the GTEx (v8) portal [50]. The *P* value was calculated by the “eQTL Calculator” tool on the GTEx (v8) official website. We only focused on protein-coding genes within 1 megabase (Mb) surrounding the association signals. The *P* value of less than 0.05 was considered to be statistically significant.

### Other analyses

Details of colocalization analyses for GWAS and eQTL signals, functional annotations of the candidate SNPs and pathway enrichment analyses are provided in the Supplementary Methods.

### Statistical analyses

The *χ*^2^ test was performed to compare the differences in clinical characteristics between the cases and controls. A fixed-effect model was used in the meta-analyses of SNPs using PLINK (v1.9) software. Cochran’s Q statistic was calculated to test the between-group heterogeneity for each SNP. The potential modification effects of age on tinnitus risk were assessed by the addition of interaction terms in the logistic regression model and by separate analyses of subgroups of subjects stratified by these factors.

## Supplementary Information

**Additional file 1: Supplementary Figure 1.** The principal components analyses (PCA) of the population in the discovery stage in this study and reference populations from the 1,000 Genomes Project. **Supplementary Figure 2.** The genotypes of rs2846071 are significantly associated with the expression levels of *WNT11* in several types of brain tissues from GTEx. **Supplementary Figure 3.** Colocalization analyses of the association signals from GWAS and brain eQTL data at the 11q13.5 and 12p13.31 loci. **Supplementary Figure 4.** Chromatin state segmentations for rs1800692 and rs4149570 using the ENCODE data. **Supplementary Figure 5.** The genotypes of rs4149577 are significantly associated with the expression levels of *TNFRSF1A* in several types of brain tissue from GTEx. **Supplementary Figure 6.** Proxy plots for 11q13.5 and 12p13.31 regions in Chinese Han Chinese and European populations. **Supplementary Figure 7.** Linkage disequilibrium plots for 11q13.5 and 12p13.31 regions in Chinese Han Chinese and European populations. **Supplementary Figure 8.** Power to detect the genetic effects of rs2846071 and rs4149577. **Supplementary Table 1.** Summary of the case/control populations used in this study. **Supplementary Table 2.** Summary of the genotyped and imputed SNPs in the discovery stage. **Supplementary Table 3.** Summary of the SNPs that have been reported to be associated with tinnitus in previous studies. **Supplementary Table 4.** Summary of the top 22 SNPs in the discovery stage. **Supplementary Table 5.** Primers used for SNPs genotyping in the replication stage. **Supplementary Table 6.** Summary of the association results in the replication stage. **Supplementary Table 7.** Stratification analyses of rs2846071 and rs4149577 by age. **Supplementary Table 8.** The predicted functional relevance of rs2846071, rs4149577 and the other SNPs in strong or moderate LD with them. **Supplementary Table 9.** Pathway analyses based on i-GSEA4GWAS. **Supplementary Table 10.** The allele and genotype frequencies of rs2846071 and rs4149577 in different populations.

## Data Availability

The datasets generated during and/or analyzed during the current study are available in the repository, http://cbportal.org/pubfiles/lyf_tinnus_298.logistic.

## Abbreviations

CI: Confidence interval
dB: decibels
eQTL: Expression quantitative trait loci
GCTA: Genome-wide Complex Trait Analysis software
GWAS: Genome-wide association study
MAF: Minor allele frequency
OR: Odds ratio
PCA: Principal component analysis
PCP: Planar cell polarity
Q-Q: Quantile-quantile
SNP: Single nucleotide polymorphism
THI: Tinnitus Handicap Inventory
TNF: Tumor necrosis factor
TNFRSF1A: Tumor necrosis factor receptor superfamily member 1A
