# Supplementary material for: Genome-wide association study identifies new loci associated with noise-induced tinnitus in Chinese populations
Source: BMC Genom Data. 2021 Sep 6;22:31. doi: 10.1186/s12863-021-00987-y (PMC8420059; doi:10.1186/s12863-021-00987-y)
Supplement: Supplementary file 1 — Additional file 1: Supplementary Figure 1. The principal components analyses (PCA) of the population in the discovery stage in this study and reference populations from the 1,000 Genomes Project. Supplementary Figure 2. The genotypes of rs2846071 are significantly associated with the expression levels of WNT11 in several types of brain tissues from GTEx. Supplementary Figure 3. Colocalization analyses of the association signals from GWAS and brain eQTL data at the 11q13.5 and 12p13.31 loci. Supplementary Figure 4. Chromatin state segmentations for rs1800692 and rs4149570 using the ENCODE data. Supplementary Figure 5. The genotypes of rs4149577 are significantly associated with the expression levels of TNFRSF1A in several types of brain tissue from GTEx. Supplementary Figure 6. Proxy plots for 11q13.5 and 12p13.31 regions in Chinese Han Chinese and European populations. Supplementary Figure 7. Linkage disequilibrium plots for 11q13.5 and 12p13.31 regions in Chinese Han Chinese and European populations. Supplementary Figure 8. Power to detect the genetic effects of rs2846071 and rs4149577. Supplementary Table 1. Summary of the case/control populations used in this study. Supplementary Table 2. Summary of the genotyped and imputed SNPs in the discovery stage. Supplementary Table 3. Summary of the SNPs that have been reported to be associated with tinnitus in previous studies. Supplementary Table 4. Summary of the top 22 SNPs in the discovery stage. Supplementary Table 5. Primers used for SNPs genotyping in the replication stage. Supplementary Table 6. Summary of the association results in the replication stage. Supplementary Table 7. Stratification analyses of rs2846071 and rs4149577 by age. Supplementary Table 8. The predicted functional relevance of rs2846071, rs4149577 and the other SNPs in strong or moderate LD with them. Supplementary Table 9. Pathway analyses based on i-GSEA4GWAS. Supplementary Table 10. The allele and genotype frequencies of rs2846071 and rs4149 [file 12863_2021_987_MOESM1_ESM.docx]

**Supplementary Material**

Genome-wide association study identifies new loci associated with noise-induced tinnitus in Chinese populations

Chengyong Xie^1^, Yuguang Niu^2^, Jie Ping^3^, Yahui Wang^3^, Chenning Yang^3^, Yuanfeng Li^3*^ and Gangqiao Zhou^1,3,4*^

^1^Medical College of Guizhou University, Guiyang city 550025, China;

^2^Department of Ambulatory Medicine, the First Medical Center of PLA General Hospital, Beijing 100853, China;

^3^State Key Laboratory of Proteomics, National Center for Protein Sciences, Beijing Institute of Radiation Medicine, Beijing 100850, China;

^4^Collaborative Innovation Center for Personalized Cancer Medicine, Center for Global Health, School of Public Health, Nanjing Medical University, Nanjing city 210029, China.

^*^Correspondence:

Dr. Gangqiao Zhou, E-mail: zhougq114@126.com;

Dr. Yuanfeng Li, E-mail: liyf_snp@163.com.

**Index**

**Supplementary methods**

**Supplementary Figures:**

**Supplementary Figure 1** The principal components analyses (PCA) of the population in the discovery stage in this study and reference populations from the 1,000 Genomes Project.

**Supplementary Figure 2** The genotypes of rs2846071 are significantly associated with the expression levels of *WNT11* in several types of brain tissues from GTEx.

**Supplementary Figure 3** Colocalization analyses of the association signals from GWAS and brain eQTL data at the 11q13.5 and 12p13.31 loci.

**Supplementary Figure 4** Chromatin state segmentations for rs1800692 and rs4149570 using the ENCODE data.

**Supplementary Figure 5** The genotypes of rs4149577 are significantly associated with the expression levels of *TNFRSF1A* in several types of brain tissue from GTEx.

**Supplementary Figure 6** Proxy plots for 11q13.5 and 12p13.31 regions in Chinese Han Chinese and European populations.

**Supplementary Figure 7** Linkage disequilibrium plots for 11q13.5 and 12p13.31 regions in Chinese Han Chinese and European populations.

**Supplementary Figure 8** Power to detect the genetic effects of rs2846071 and rs4149577.

**Supplementary Tables:**

**Supplementary Table 1** Summary of the case/control populations used in this study.

**Supplementary Table 2** Summary of the genotyped and imputed SNPs in the discovery stage.

**Supplementary Table 3** Summary of the SNPs that have been reported to be associated with tinnitus in previous studies.

**Supplementary Table 4** Summary of the top 22 SNPs in the discovery stage.

**Supplementary Table 5** Primers used for SNPs genotyping in the replication stage.

**Supplementary Table 6** Summary of the association results in the replication stage.

**Supplementary Table 7** Stratification analyses of rs2846071 and rs4149577 by age.

**Supplementary Table 8** The predicted functional relevance of rs2846071, rs4149577 and the other SNPs in strong or moderate LD with them.

**Supplementary Table 9** Pathway analyses based on i-GSEA4GWAS.

**Supplementary Table 10** The allele and genotype frequencies of rs2846071 and rs4149577 in different populations.

**Supplementary methods**

**Colocalization analyses for GWAS and eQTL signals**

To provide colocalization evidence for the GWAS and eQTL signals at the 11q13.5 and 12p13.31 loci, we performed colocalization analyses using the R package “Coloc” (3.2.1). Evidence for colocalization was assessed using the posterior probability (PP) for five exclusive hypotheses: H0, neither trait has a genetic association in the region; H1/H2, only one trait has a genetic association in the region; H3, both traits are associated but with different causal variants; and H4, both traits are associated and share a single causal variant. Associations with a posterior probability of hypothesis 4 (PP4) > 0.8 were deemed to be ‘highly likely to colocalize’. Associations with a PP4 > 0.2 indicated suggestive evidence of sharing the same variant with GWAS and eQTL signals. Colocalization analysis plots were generated using the online tool LocusCompare (<http://locuscompare.com/>). The eQTL data for rs2846071-*WNT11* in brain cerebellum tissues and rs4149577-*TNFRSF1A* in brain caudate tissues were downloaded from the GTEx (v8). We used eQTL data in these tissues because the most significant eQTL signals for rs2846071-*WNT11* and rs4149577-*TNFRSF1A* were detected in these tissues, respectively. The GWAS data for rs2846071 and rs4149577 were extracted from the present study. We then used these eQTL data and GWAS data to perform colocalization analyses. The rs2846071 was not colocalized with eQTL signals for *WNT11* in brain tissues, as the lead eQTL variant for *WNT11* (rs71469506, *P*_eQTL_ = 2.50 × 10^-4^) shows low LD (*r*^2^ < 0.2) with the rs2846071. The SNP rs4149577 was colocalized with eQTL signals for *TNFRSF1A* in brain tissues, as the lead eQTL variant for *TNFRSF1A* (rs3481970, *P*_eQTL_ = 1.38 × 10^-3^) shows mild LD (*r*^2^ > 0.2) with the rs4149577.

**Functional annotations of the candidate SNPs**

Functional annotations of SNPs were performed using multiple tools, including the HaploReg (v4.1)[1] and Probabilistic Annotation Integrator (PAINTOR) (v3.0) [2]. HaploReg (<http://pubs.broadinstitute.org/mammals/haploreg/haploreg.php>) is a resource for the annotation of chromatin states, conservation and regulatory motifs for human SNPs.[1] PAINTOR is a novel fine-mapping method, which uses a Bayesian method to determine the SNPs with the highest probability of causality. We set the following parameters for PAINTOR method: enumerate was set to 3, and three types of DNA elements (coding, enhancer and DNase I hypersensitive site [DHS]) were involved in the calculation of posteriori probabilities in the function annotation file. The method integrates the *P* value of the association, LD structure and functional annotation data to calculate the causality posterior probability for each SNP.

**Pathway enrichment analyses**

Pathway enrichment analyses were performed to explore the potential biologically relevant pathways involved in tinnitus, using the improved gene-set-enrichment analysis approach (i-GSEA4GWAS) (v1) [3]. The whole-genome SNPs and their *P* values were input into the i-GSEA4GWAS. To avoid the overrepresentation of SNPs in more than one gene, we restricted mapping SNPs to +/-20 kilobases (kb) around a gene. Then, the canonical pathway of gene-sets was used for further analysis, which was extracted and curated from the Molecular Signatures Database (MSigDB) [4]. Then, i-GSEA4GWAS performs SNP-label permutations to assess the significance of the pathway-based enrichment score and to give the false discovery rate (FDR) for multiple testing corrections. The FDR of less than 0.1 was considered to be statistically significant. This threshold value was widely used in previous reports [5].


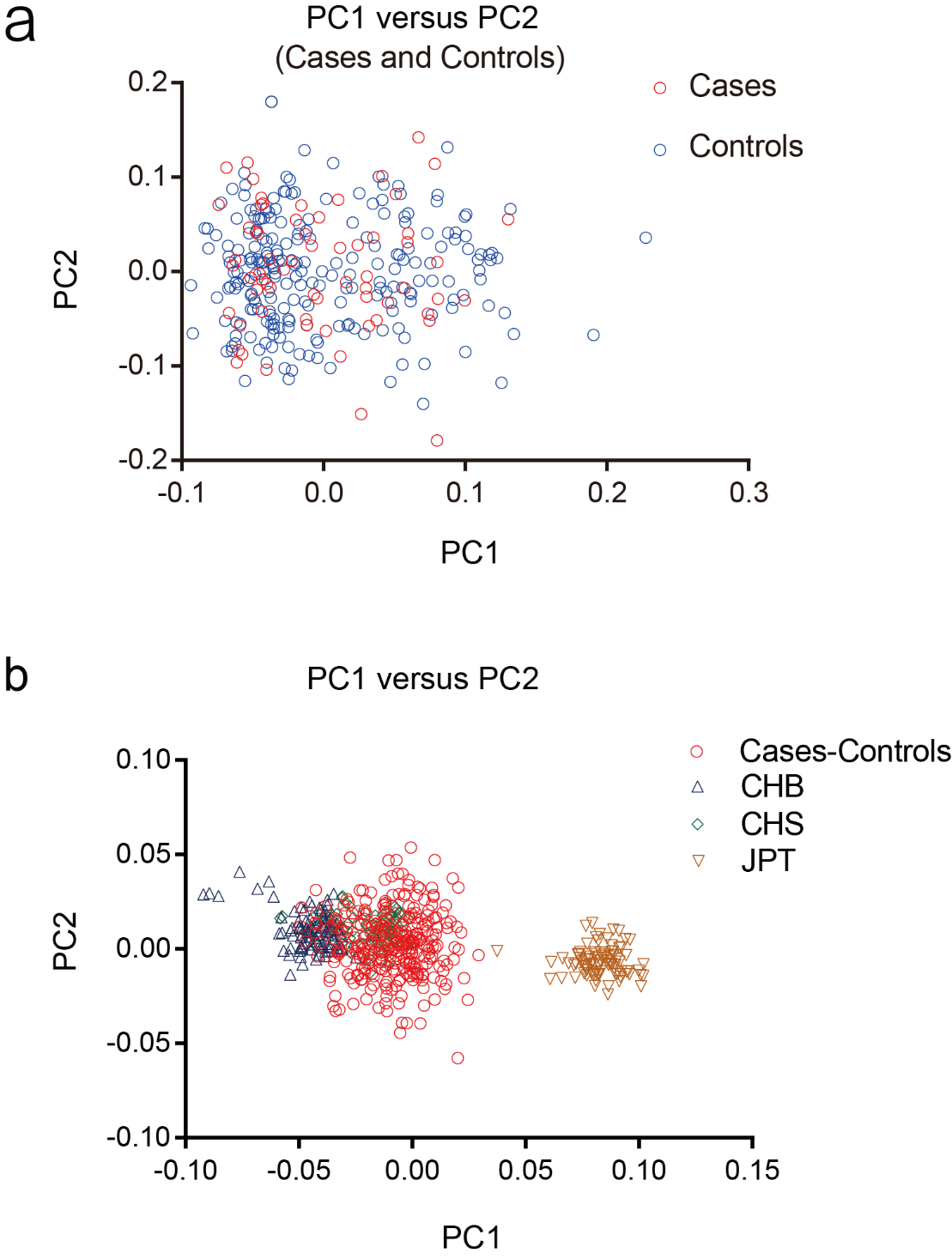


**Supplementary Fig. 1 The principal components analyses (PCA) of the population in the discovery stage in this study and reference populations from the 1,000 Genomes Project.** (**a**) Principal component (PC) 1 versus PC2 for the 65 cases (red) and the 233 controls (blue) in the discovery stage in this study. (**b**) PC1 versus PC2 for the subjects in the discovery stage (red) and the reference individuals in the 1,000 Genomes Project (phase 3; n = 282), including 93 CHBs (Han Chinese in Beijing, China; blue), 100 CHSs (Southern Han Chinese, China; green) and 89 JPTs (Japanese in Tokyo, Japan; brown).

**
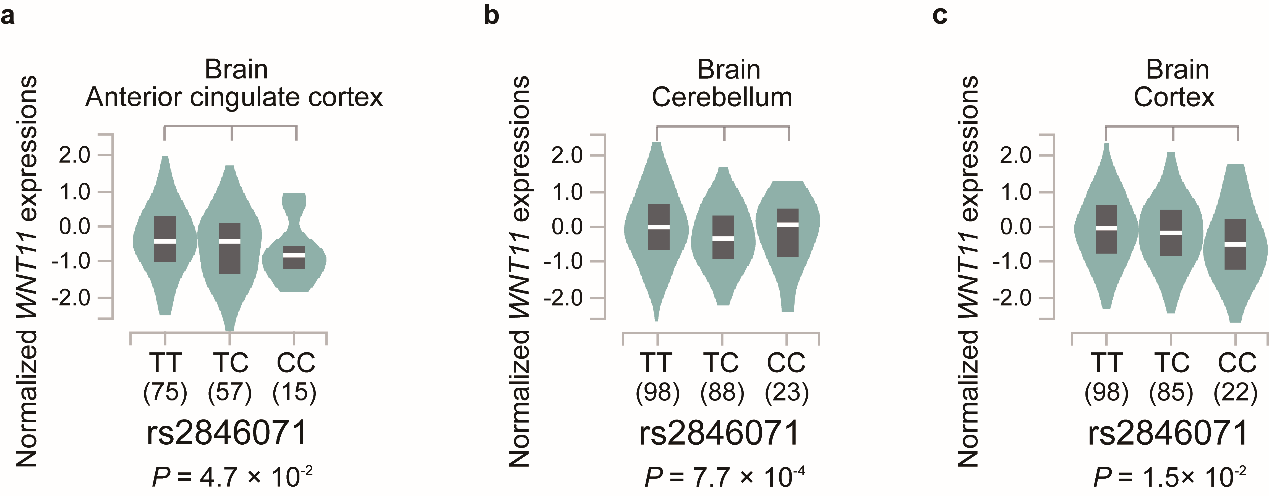
**

**Supplementary Fig. 2 The genotypes of rs2846071 are significantly associated with the expression levels of *WNT11* in several types of brain tissues from GTEx.** The expression levels of *WNT11* based on different rs2846071 genotypes (TT, TC and CC) in the brain anterior cingulate cortex (**a**), cerebellum (**b**) and cortex (**c**) tissues were shown. The data of *WNT11* mRNA expression levels were derived from the GTEx. The *WNT11* mRNA expression levels were normalized by the trimmed mean of M-values (TMM) approach and were then log2 transformed. The number below the genotype indicates the number of samples in each genotype. *P* value was generated using a linear regression model between the genotypes and expression levels, and *P* value less than 0.05 was considered to be statistically significant. However, multiple testing corrections may be used to avoid false-positive results. After Bonferroni multiple testing correction, rs2846071 genotypes were only significantly associated with *WNT11* mRNA expressions in the brain cerebellum (*P* < 3.8 × 10^-3^, *i.e.*, 0.05/13 types of brain tissue). The white line in the box plot (black) shows the median value of the expression levels of each genotype.

**
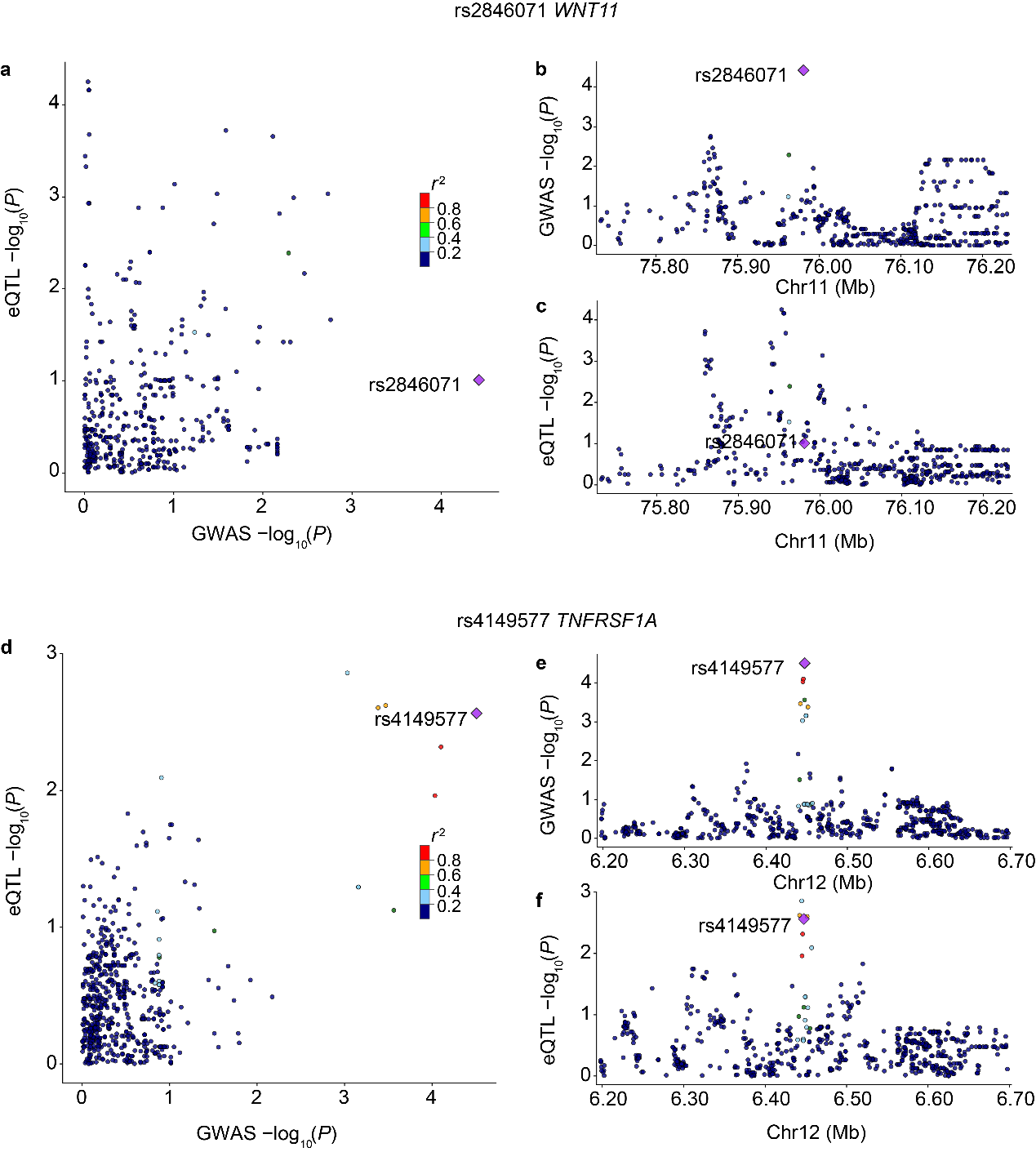
**

**Supplementary Fig. 3 Colocalization analyses of the association signals from GWAS and brain eQTL data at the 11q13.5 and 12p13.31 loci.** LocusCompare plots for *WNT11* (**a-c**) and *TNFRSF1A* (**d-f**) on 11q13.5 and 12p13.31 were shown. Genomic positions are based on NCBI Build 37. The *P* values of rs2846071 and rs4149577 were shown as purple diamonds. The linkage disequilibrium (LD) values (*r*^2^) to rs2846071 and rs4149577 for the other SNPs are indicated by marked color. Red signifies *r*^2^ > 0.8, orange 0.6 < *r*^2^ ≤ 0.8, green 0.4 < *r*^2^ ≤ 0.6, light blue 0.2 < *r*^2^ ≤ 0.4 and blue *r*^2^ ≤ 0.2. Associations with a posterior probability of hypothesis 4 (PP4) (*i.e.*, both traits are associated and share a single causal variant) > 0.8 was deemed to be “highly likely to colocalize”. Associations with a PP4 > 0.2 indicated suggestive evidence of sharing the same variant with GWAS and eQTL signals. At 11q13.5, the colocalization analyses based on eQTL data of the brain cerebellum showed that the PP4 was only 0.025, which indicated that the tinnitus-associated SNP rs2846071 was not colocalized with eQTL signals for *WNT11* in brain tissues. At 12p13.31, the colocalization analyses based on eQTL data of the brain caudate showed that the PP4 was 0.288, suggesting that the tinnitus-associated SNP rs4149577 is colocalized with eQTL signals for *TNFRSF1A* in brain tissues.


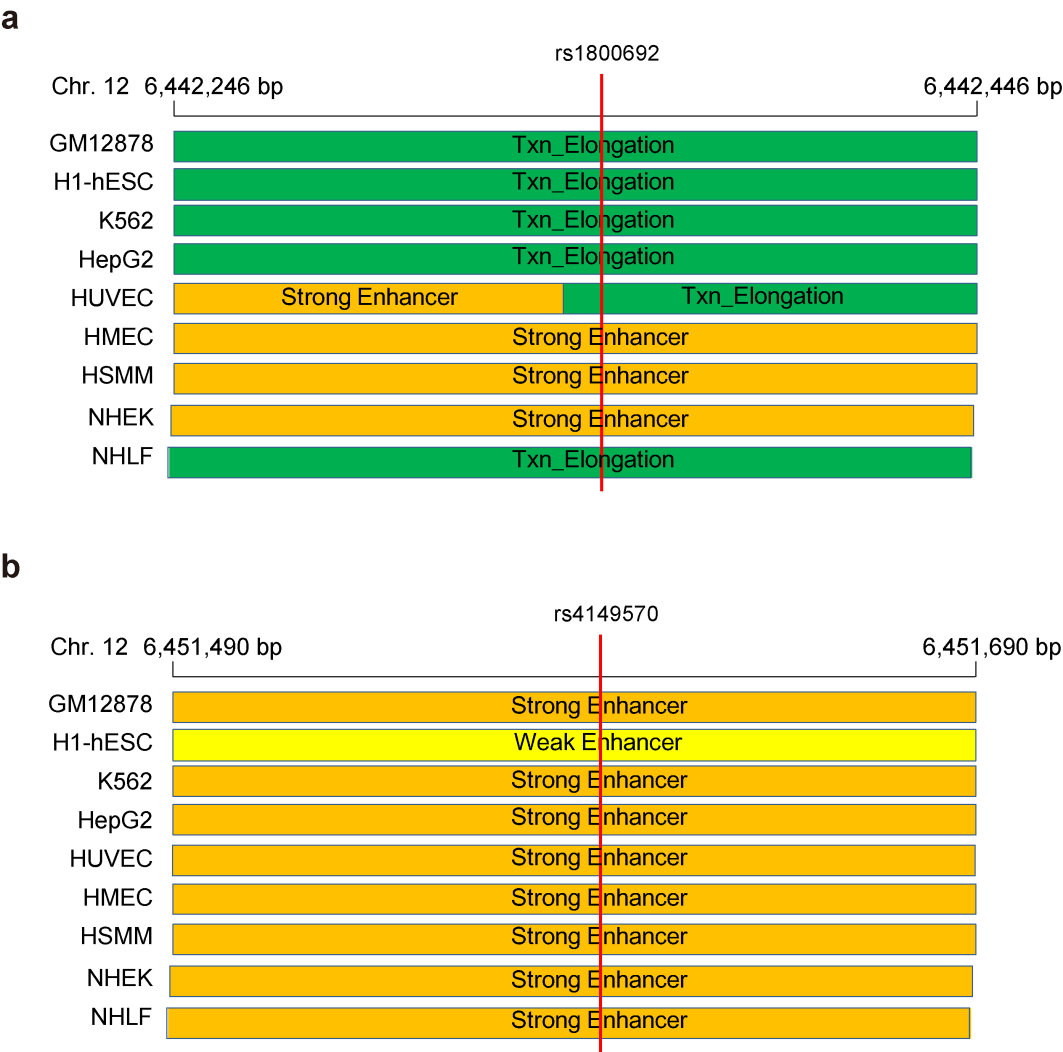


**Supplementary Fig. 4 Chromatin state segmentations for rs1800692 and rs4149570 using the ENCODE data.** Functional annotations were performed using the data from the Encyclopedia of DNA Elements (ENCODE) project. The epigenetic signals evaluated include histone modifications. All tracks were generated by the UCSC genome browser (The UCSC genome browser; http://genome.ucsc.edu; hg19). Positions of rs1800692 and rs4149570 are highlighted with red lines.

**
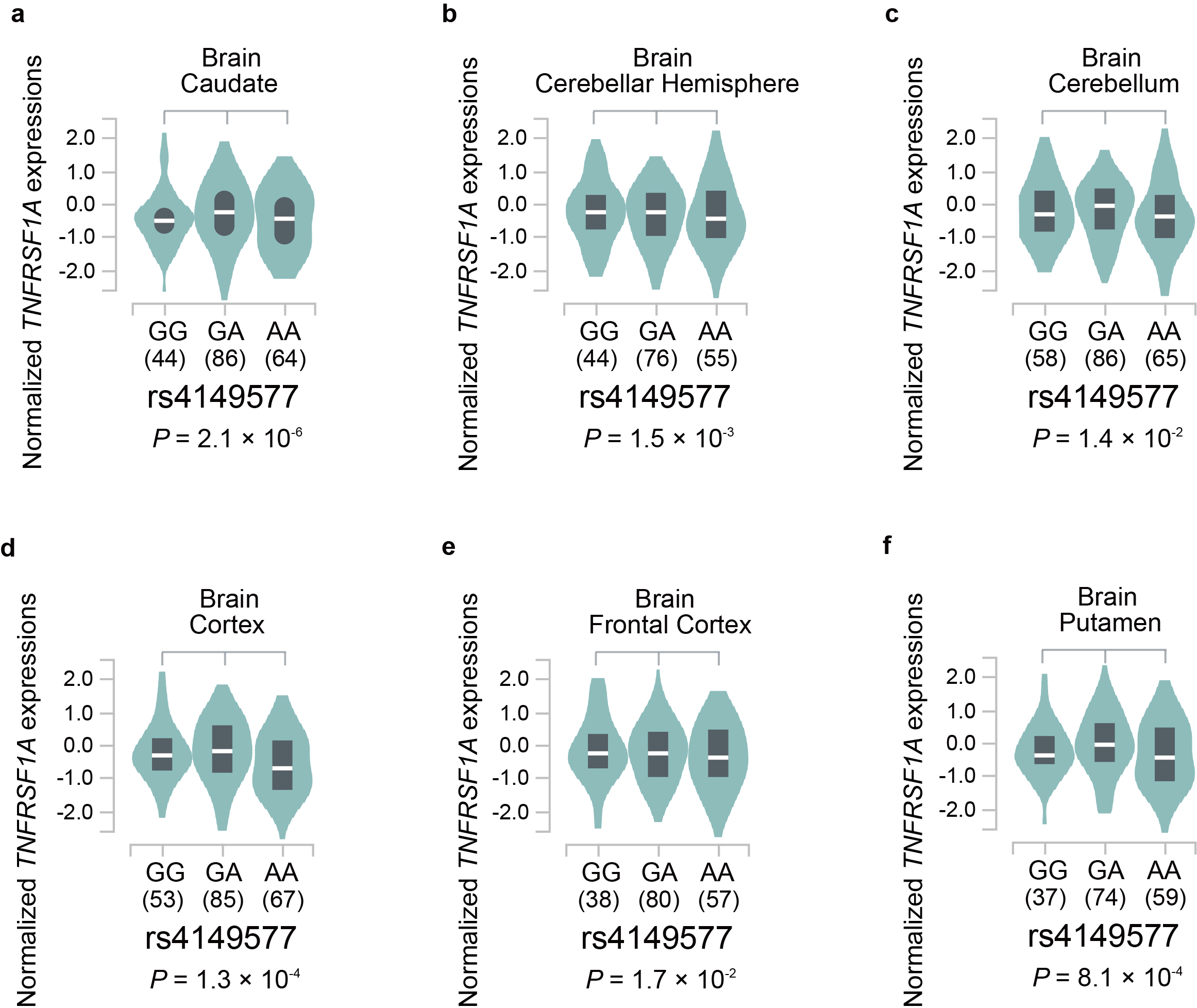
Supplementary Fig. 5 The genotypes of rs4149577 are significantly associated with the expression levels of *TNFRSF1A* in several types of brain tissue from GTEx.** The expression levels of *TNFRSF1A* on the basis of different rs4149577 genotypes (GG, GA and AA) in the brain caudate (a), cerebellar hemisphere (b), cerebellum (c), cortex (d), frontal cortex (e) and putamen (f) tissues were shown. The data of *TNFRSF1A* mRNA expression levels were derived from GTEx. The *TNFRSF1A* mRNA expression levels were normalized by the trimmed mean of M-values (TMM) approach and were then log2 transformed. The number below the genotype indicates the number of samples in each genotype. *P* value was generated using a linear regression model between the genotypes and expression levels, and a *P* value less than 0.05 was considered to be statistically significant. However, multiple testing corrections may be used to avoid false-positive results. After Bonferroni multiple testing correction, rs4149577 genotypes remained to be significantly associated with *TNFRSF1A* mRNA expressions in brain caudate, brain cerebellar hemisphere, brain cortex and brain putamen (*P* < 3.8 × 10^-3^, *i.e.*, 0.05/13 types of brain tissue). The white line in the box plot (black) shows the median value of the expression levels of each genotype.


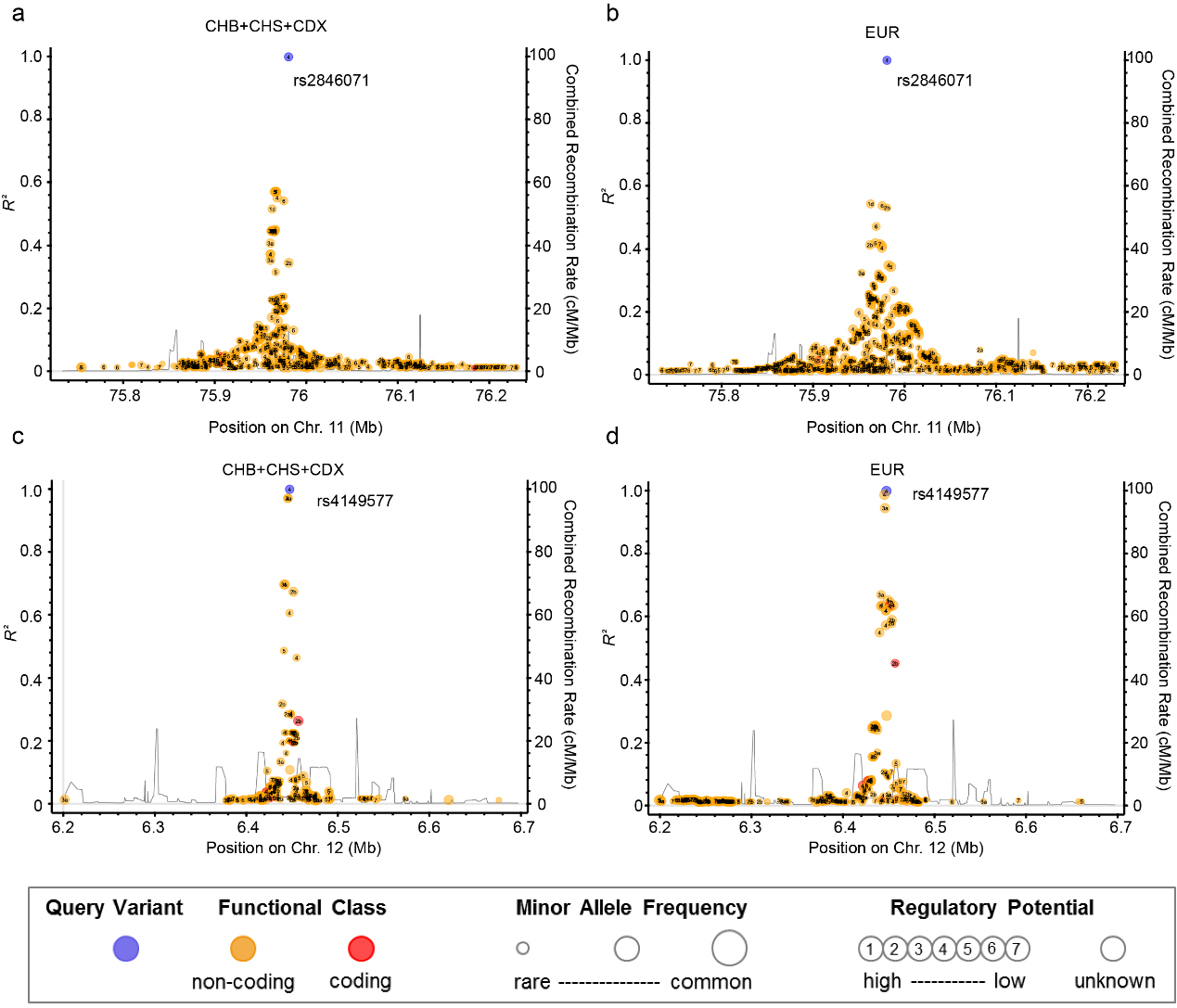
**Supplementary Fig. 6 Proxy plots for 11q13.5 and 12p13.31 regions in Chinese Han Chinese and European populations.** Proxy plots for 11q13.5 (**a-b**) and 12p13.31 (**c-d**) were shown. The proxy plots were plotted using LDlink (https://ldlink.nci.nih.gov/). Genomic positions are based on NCBI Build 37. Phase 3 haplotype data from the 1000 Genomes Project are referenced for calculating pairwise metrics of linkage disequilibrium (LD), searching for proxies in high LD. The rs2846071 and rs4149577 were shown as purple dots. The LD values (*r*^2^) to rs2846071 and rs4149577 for the other SNPs are indicated by the y-axis. The x-axis represents the genomic positions. Chinese Han populations include Chinese Dai in Xishuangbanna, China (CDX), Han Chinese in Beijing, China (CHB), Han Chinese South (CHS). European populations (EUR) include the British in England and Scotland (GBR), Western European ancestry (CEU), Finnish in Finland (FIN), Iberian Population in Spain (IBS) and Toscani in Italia (TSI). Chr., chromosome; megabase, Mb.

**
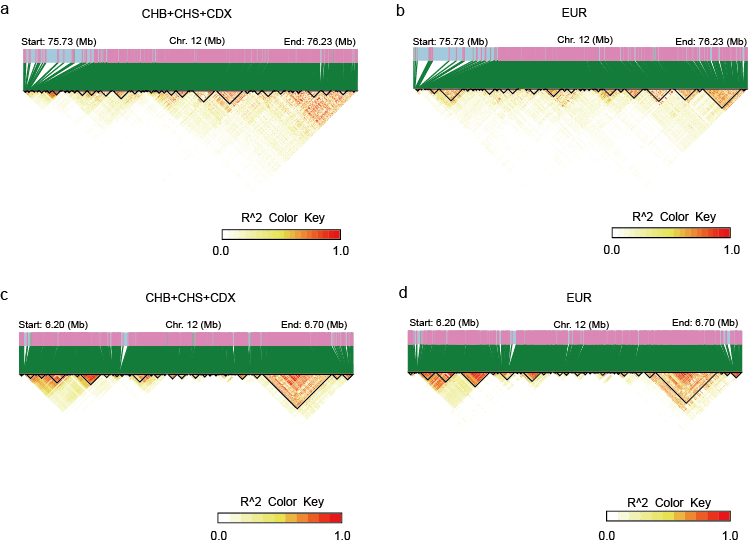
**

**Supplementary Fig. 7** **Linkage disequilibrium plots for 11q13.5 and 12p13.31 regions in Chinese Han Chinese and European populations.** The linkage disequilibrium plots in the 500 kb region of index SNP rs2846071 (**a-b**) and rs4149577 (**c-d**) were plotted using LDBlockShow (https://github.com/BGI-shenzhen/LDBlockShow). Genomic positions are based on NCBI Build 37. Phase 3 haplotype data from the 1000 Genomes Project are referenced for calculating linkage disequilibrium (LD) blocks. Chinese Han populations include Chinese Dai in Xishuangbanna, China (CDX), Han Chinese in Beijing, China (CHB), Han Chinese South (CHS). European populations (EUR) include the British in England and Scotland (GBR), Western European ancestry (CEU), Finnish in Finland (FIN), Iberian Population in Spain (IBS) and Toscani in Italia (TSI). Chr., chromosome; megabase, Mb.


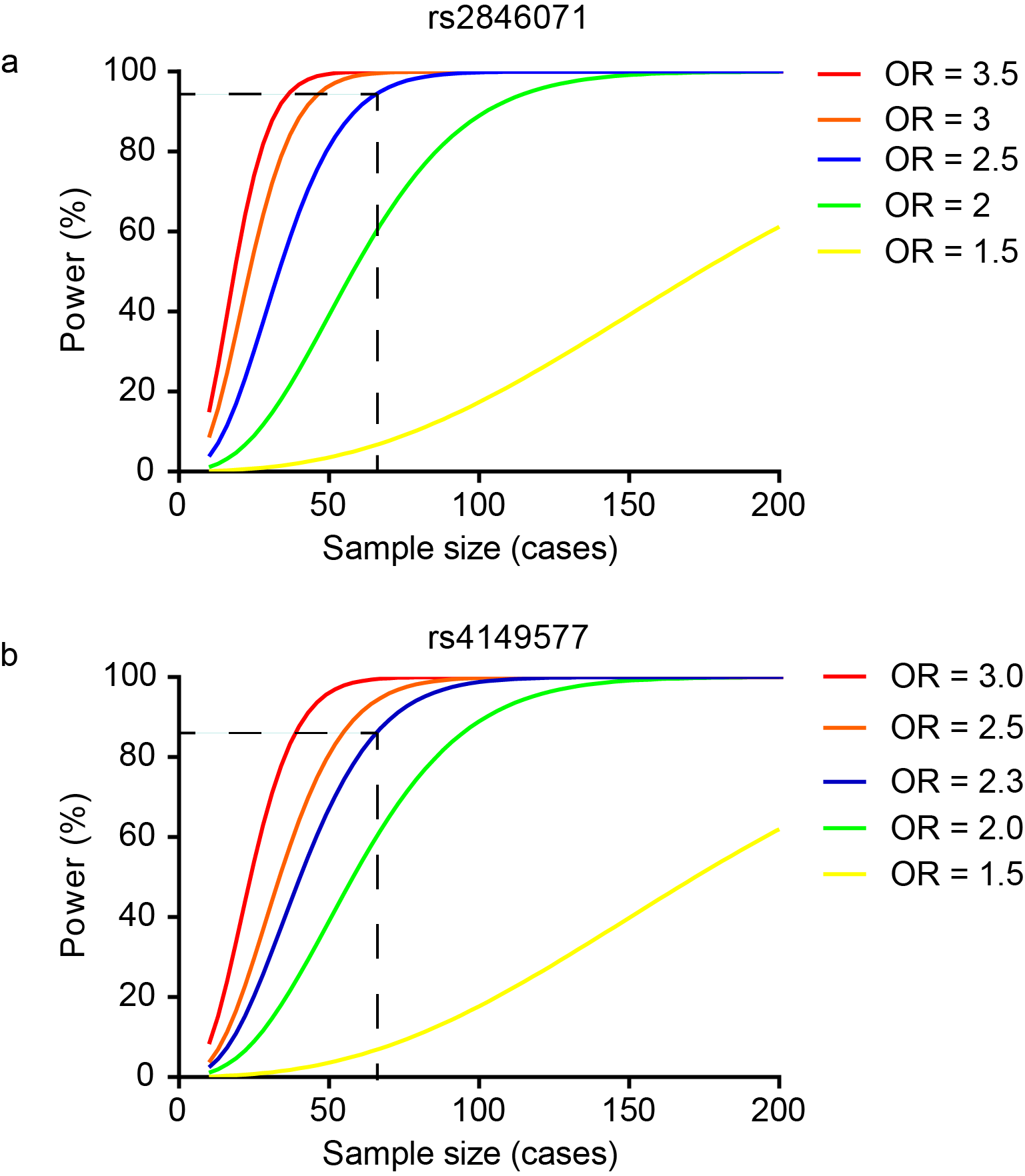


**Supplementary Fig. 8 Power to detect the genetic effects of rs2846071 and rs4149577.** Power is reported here as the probability of SNPs to be identified in a scan. Vertical and horizontal dashed lines show that the power of rs2846071 (**a**) and rs4149577 (**b**), given tinnitus prevalence of 15%, 65 cases and 233 controls, and the allele frequencies of 0.341 and 0.359, respectively. OR, odds ratio.

**Supplementary Table 1 Summary of the case/control populations used in this study.**

| Categories | Discovery stage | | *P* | Replication stage | | *P* | Overall | | *P* |
| --- | --- | --- | --- | --- | --- | --- | --- | --- | --- |
|  | Cases  (n = 65) | Controls  (n = 233) |  | Cases  (n = 34) | Controls  (n = 379) |  | Cases  (n = 99) | Controls  (n = 612) |  |
| Age, years |  |  |  |  |  |  |  |  |  |
| Mean (SD) | 23.8 (1.6) | 23.4 (1.6) | 0.080 | 26.4 (3.9) | 24.5 (2.9) | 0.0070 | 24.7 (2.7) | 24.1 (2.7) | 0.075 |
| ≤ 23, n (%) | 25 (38.5) | 113 (48.5) | 0.15 | 7 (20.6) | 141 (37.2) | 0.053 | 32 (32.3) | 254 (41.5) | 0.084 |
| > 23, n (%) | 40 (61.5) | 120 (51.5) |  | 27 (79.4) | 238 (62.8) |  | 67 (67.7) | 358 (58.5) |  |
| Gender, n (%) |  |  |  |  |  |  |  |  |  |
| Male | 65 (100) | 233 (100) |  | 34 (100) | 379 (100) |  | 99 (100) | 612 (100) |  |
| Female | 0 (0) | 0 (0) |  | 0 (0) | 0 (0) |  | 0 (0) | 0 (0) |  |
| Noise exposure time, years |  |  |  |  |  |  |  |  |  |
| Mean (SD) | 4.6 (0.96) | 4.4 (1.07) | 0.20 | 2.8 (2.41) | 2.2 (1.94) | 0.36 | 3.4 (2.31) | 3.0 (1.47) | 0.010 |
| SD, standard deviation. | | | | | | |  |  |  |

**Supplementary Table 2 Summary of the genotyped and imputed SNPs in the discovery stage.**

| Chr. | No. of genotyped SNPs passed QC | No. of SNPs after imputed | No. of imputed SNPs passed QC |
| --- | --- | --- | --- |
| 1 | 24,868 | 6,500,406 | 309,001 |
| 2 | 24,730 | 7,117,653 | 328,153 |
| 3 | 21,516 | 5,862,644 | 289,315 |
| 4 | 20,116 | 5,763,688 | 292,591 |
| 5 | 18,091 | 5,293,987 | 244,850 |
| 6 | 23,424 | 5,051,726 | 289,036 |
| 7 | 17,225 | 4,741,633 | 240,298 |
| 8 | 10,893 | 4,622,584 | 152,737 |
| 9 | 8,290 | 3,576,648 | 103,796 |
| 10 | 16,465 | 4,013,474 | 212,790 |
| 11 | 15,392 | 4,067,172 | 207,977 |
| 12 | 14,730 | 3,889,071 | 192,519 |
| 13 | 10,872 | 2,872,976 | 143,587 |
| 14 | 10,136 | 2,650,950 | 128,413 |
| 15 | 10,083 | 2,437,496 | 110,005 |
| 16 | 10,442 | 2,713,904 | 111,854 |
| 17 | 9,716 | 2,341,798 | 97,318 |
| 18 | 9,413 | 2,279,220 | 109,551 |
| 19 | 7,989 | 1,843,244 | 84,304 |
| 20 | 7,999 | 1,822,234 | 79,851 |
| 21 | 4,965 | 1,104,664 | 54,386 |
| 22 | 4,898 | 1,110,227 | 48,099 |
| Sum | 302,253 | 81,677,399 | 3,830,431 |
| Chr., chromosome; QC, quality control; SNP, single nucleotide polymorphism; No., number. Imputation on the GWAS data sets was performed using the IMPUTE2 software (version 2.3.1), on the basis of the 1,000 Genomes Project data (phase 3). | | | |
|  |  |  |  |
|  |  |  |  |

**Supplementary Table 3 Summary of the SNPs that have been reported to be associated with tinnitus in previous studies.**

| No. | SNPs | Positions^a^ | Genes^b^ | PMID | Diseases | Sample sizes | Ancestries | ORs (95% CIs)^c^ | *P* | In the present study | |
| --- | --- | --- | --- | --- | --- | --- | --- | --- | --- | --- | --- |
|  |  |  |  |  |  |  |  |  |  | ORs (95% CIs)^c^ | *P* |
| 1 | rs12026039 | 104571091 | *AL136455.1 - AC092506.1* | 30952644 | DIT | 762 | European | 3.52 (2.99 - 4.06) | 4.0E-6 | 1.22 (0.81 - 1.83) | 0.342 |
| 2 | rs10842948 | 27749042 | *PPFIBP1* | 30952644 | DIT | 762 | European | 2.48 (2.09 - 2.88) | 5.0E-6 | NA | NA |
| 3 | rs7532231 | 104736067 | *AC092506.1* | 30952644 | DIT | 762 | European | 2.21 (1.89 - 2.53) | 1.0E-6 | 0.85 (0.56 - 1.29) | 0.447 |
| 4 | rs6552561 | 183293516 | *TENM3* | 30952644 | DIT | 762 | European | 1.85 (1.58 - 2.12) | 9.0E-6 | 1.02 (0.57 - 1.82) | 0.952 |
| 5 | rs498518 | 234830590 | *AL160408.3* | 30952644 | DIT | 762 | European | 1.86 (1.59 - 2.14) | 9.0E-6 | 1.38 (0.73 - 2.60) | 0.317 |
| 6 | rs73231578 | 26681200 | *ADRA1A* | 30952644 | DIT | 762 | European | 2.94 (2.48 - 3.40) | 4.0E-6 | NA | NA |
| 7 | rs117764890 | 31465897 | *LINC02664* | 30952644 | DIT | 762 | European | 3.42 (2.89 - 3.95) | 6.0E-6 | 0.57 (0.22 - 1.34) | 0.185 |
| 8 | rs6671895 | 38555026 | *AL139158.2* | 30952644 | DIT | 762 | European | 4.32 (3.73 - 4.91) | 1.0E-6 | 0.77 (0.33 - 1.81) | 0.548 |
| 9 | rs141382055 | 22859811 | *PIP4K2A* | 30952644 | DIT | 762 | European | 5.70 (4.97 - 6.43) | 3.0E-6 | NA | NA |
| 10 | rs1377817 | 50804066 | *MYH14* | 31296530 | DIT | 866 | European | NA | 4.6E-8 | NA | NA |
| 11 | rs1110149 | 37824053 | *GDNF* | 27180191 | CT | 52 | European | NA | 2.0E-2 | 1.40 (0.92 - 2.13) | 0.117 |
| 12 | rs915539 | 35829070 | *KCNE1* | 23224734 | NIT | 626 | European | NA | 5.0E-3 | 1.00 (1.00 - 1.00) | NA |
| 13 | rs10089 | 127522543 | *SLC12A2* | 22654739 | NIT | 95 | European | NA | 1.6E-2 | 0.85 (0.57 - 1.27) | 0.430 |
| 14 | rs34544607 | 77459750 | *KCTD12* | 28533738 | CT | 95 | European | NA | 4.0E-2 | 1.00 (1.00 - 1.00) | NA |
| 15 | rs6265 | 27679916 | *BDNF* | 33685039 | CT | 338 | East Asians | NA | 5.0E-2 | 0.93 (0.64 - 1.37) | 0.720 |
| 16 | rs4947296 | 31058178 | *U6* | 29326686 | MD | 2,050 | European | 2.09 (1.66 - 2.63) | 1.4E-9 | 0.89 (0.51 - 1.57) | 0.700 |
| 17 | rs4788863 | 73089852 | *SLC16A5* | 28448657 | Oto | 188 | European | 0.060 (0.020 - 0.22) | 2.2E-7 | 1.20 (0.82 - 1.81) | 0.370 |
| 18 | rs4646316 | 19952132 | *COMT* | 28445188 | Oto | 149 | European | 1.50 (1.15 - 1.95) | NA | 1.23 (0.83 - 1.84) | 0.310 |
| 19 | rs1872328 | 54395259 | *ACYP2* | 28445188 | Oto | 149 | European | 5.91 (1.51 - 23.16) | NA | NA | NA |
| 20 | rs1695 | 67352689 | *GSTP1* | 27729156 | Oto | 72 | European | NA | NA | 1.24 (0.80 - 1.94) | 0.340 |
| 21 | rs4880 | 160113872 | *SOD2* | 26400460 | Oto | 71 | European | 0.36 (1.30 - 7.20) | NA | 0.66 (0.35 - 1.26) | 0.210 |
| 22 | rs2075252 | 170010985 | *LRP2* | 17457342 | Oto | 50 | European | 3.45 (1.11 - 11.20) | 2.0E-2 | 1.25 (0.83 - 1.89) | 0.280 |
| 23 | rs4906228 | 103050764 | *RCOR1* | 33742053 | Tinnitus | 172,608 | European | 1.08 (1.05 - 1.00) | 1.7E-8 | NA | NA |

CI, confidence interval; CT, chronic tinnitus; DIT, drug-induced tinnitus; MD, Meniere's disease; NIT, noise-induced tinnitus; GWAS, genome-wide association analysis; NA, not available; OR, odds ratio; SNP, single nucleotide polymorphism; Oto, Ototoxicity. ^a^Positions based on NCBI Build 37; ^b^The nearest genes; ^c^ORs and 95% CIs were calculated for the minor allele.

**Supplementary Table 4 Summary of the top 22 SNPs in the discovery stage.**

| No. | Chr. | SNPs | Positions^a^ | Alleles^b^ | A1 hom / Het / A2 hom^c^ | | | Genotyped or  imputed^d^ | Genes^e^ | ORs (95% CIs)^f^ | *P* |
| --- | --- | --- | --- | --- | --- | --- | --- | --- | --- | --- | --- |
|  |  |  |  |  | Cases-controls | Cases | Controls |  |  |  |  |
| 1 | 2 | rs2540334 | 202271347 | T/C | 24/96/178 | 15/21/29 | 9/75/149 | G | *TRAK2* | 2.46 (1.62-3.74) | 2.67E-05 |
| 2 | 2 | rs13030006 | 206666066 | T/C | 47/142/109 | 16/39/10 | 31/103/99 | G | *NRP2* | 2.48 (1.61-3.82) | 4.05E-05 |
| 3 | 4 | rs6812414 | 38322807 | A/C | 6/73/219 | 4/25/36 | 2/48/183 | G | *RP11-600L4.1* | 2.82 (1.68-4.74) | 9.16E-05 |
| 4 | 4 | rs4692983 | 94392039 | G/T | 13/113/169 | 6/35/23 | 7/78/146 | I | *GRID2* | 2.65 (1.64-4.27) | 6.60E-05 |
| 5 | 4 | rs11936923 | 161091459 | A/T | 9/64/213 | 6/21/35 | 3/43/178 | I | *RP11-502M1.2* | 2.83 (1.71-4.69) | 5.30E-05 |
| 6 | 5 | rs34607327 | 54001948 | T/A | 1/35/241 | 1/16/42 | 0/19/199 | I | *AC112198.2* | 4.68 (2.22-9.86) | 5.01E-05 |
| 7 | 6 | rs9350458 | 73133122 | A/T | 43/133/96 | 16/35/7 | 27/98/89 | I | *RIMS1* | 2.74 (1.73-4.35) | 1.85E-05 |
| 8 | 7 | rs17406935 | 78664922 | A/G | 1/42/255 | 1/20/44 | 0/22/211 | G | *MAGI2* | 4.57 (2.33-8.97) | 1.01E-05 |
| 9 | 7 | rs1188974 | 158665748 | A/G | 3/35/260 | 3/15/47 | 0/20/213 | G | *WDR60* | 4.10 (2.09-8.03) | 4.05E-05 |
| 10 | 8 | rs660641 | 2873709 | C/T | 12/102/183 | 7/30/28 | 5/72/155 | G | *CSMD1* | 2.67 (1.66-4.30) | 5.63E-05 |
| 11 | 9 | rs11141939 | 90290400 | G/T | 26/124/144 | 14/30/21 | 12/94/123 | I | *DAPK1* | 2.39 (1.56-3.69) | 7.34E-05 |
| 12 | 10 | rs2296466 | 23393091 | G/A | 9/91/198 | 6/28/31 | 3/63/167 | G | *MSRB2* | 2.63 (1.62-4.29) | 9.90E-05 |
| 13 | 11 | rs2846071 | 75980710 | T/C | 30/143/125 | 14/36/15 | 16/107/110 | G | *WNT11* | 2.54 (1.63-3.96) | 3.75E-05 |
| 14 | 11 | rs72983568 | 102773998 | G/T | 0/35/238 | 0/19/42 | 0/16/196 | I | *U7* | 5.59 (2.64-11.84) | 7.14E-06 |
| 15 | 12 | rs4149577 | 6447522 | A/G | 45/124/129 | 19/31/15 | 26/93/114 | G | *TNFRSF1A* | 2.33 (1.56-3.46) | 3.09E-05 |
| 16 | 12 | rs10771523 | 9472268 | A/G | 3/64/204 | 3/24/34 | 0/40/170 | I | *SNORA75* | 3.67 (2.05-6.58) | 1.24E-05 |
| 17 | 15 | rs1551465 | 101527412 | G/A | 62/149/85 | 5/29/30 | 57/120/55 | G | *LRRK1* | 0.41 (0.26-0.64) | 7.17E-05 |
| 18 | 17 | rs3851793 | 45059900 | T/C | 12/128/158 | 8/34/23 | 4/94/135 | G | *GOSR2* | 2.68 (1.65-4.36) | 7.11E-05 |
| 19 | 17 | rs7224395 | 67653207 | T/C | 37/146/95 | 15/39/8 | 22/107/87 | I | *AC003051.1* | 2.78 (1.74-4.42) | 1.74E-05 |
| 20 | 18 | rs148091530 | 12419210 | G/A | 3/39/231 | 3/16/38 | 0/23/193 | I | *SLMO1* | 3.99 (2.05-7.78) | 4.75E-05 |
| 21 | 19 | rs79246064 | 13317457 | C/T | 1/34/262 | 1/16/47 | 0/18/215 | G | *CACNA1A* | 4.70 (2.24-9.84) | 4.08E-05 |
| 22 | 20 | rs4402823 | 47089525 | T/C | 66/151/73 | 3/36/25 | 63/115/48 | G | *RP5-991B18.1* | 0.39 (0.25-0.60) | 2.81E-05 |

CI, confidence interval; Chr., chromosome; OR, odds ratio; SNP, single-nucleotide polymorphism. ^a^Positions based on GRCh37; ^b^Minor allele/major allele; ^c^Counts of Minor allele homozygote/heterozygote/major allele homozygote; ^d^Types of SNPs: “G” stands for the genotyped SNPs, and “I” stands for the imputed SNPs; ^e^Genes or nearest genes; ^f^ORs and 95% CIs were calculated for the minor allele.

**Supplementary Table 5 Primers used for SNPs genotyping in the replication stage.**

| No. | SNPs | Genotyped or  imputed^a^ | Primers | Sequences (5'→3') |
| --- | --- | --- | --- | --- |
| 1 | rs2540334 | G | Forward | ACGTTGGATGAAATGTCCAGGATCTGATGC |
|  |  |  | Reverse | ACGTTGGATGGTGTGCACAGTAGCTGTTAG |
|  |  |  | Extend | CCCCGGATCTGATGCAATCACCTA |
| 2 | rs13030006 | G | Forward | ACGTTGGATGGAAGAGTCCTGGAAGCTTAC |
|  |  |  | Reverse | ACGTTGGATGTGGCTCAAGAGTTCTGAGAG |
|  |  |  | Extend | GGAAGCTTACAGGTCG |
| 3 | rs6812414 | G | Forward | ACGTTGGATGTGGACCCACTAAGAGAGATG |
|  |  |  | Reverse | ACGTTGGATGCAATGATACACAGGTATCATC |
|  |  |  | Extend | TATTCGGGGAAATCCCGTGA |
| 4 | rs4692983 | I | Forward | ACGTTGGATGAACAGTCTTATTAAGAGCC |
|  |  |  | Reverse | ACGTTGGATGTATCACTAAGAATGAGCCAC |
|  |  |  | Extend | AGCCATTTTCATATAAAATAGTACATT |
| 5 | rs11936923 | I | Forward | ACGTTGGATGCAAGGTAAGGTTTCATAGAC |
|  |  |  | Reverse | ACGTTGGATGTGGCATACAGATGCTTTTC |
|  |  |  | Extend | AATAGACTAAGGACTTAGATTTTC |
| 6 | rs34607327 | I | Forward | ACGTTGGATGTGACTCTGAGATGTACCAGC |
|  |  |  | Reverse | ACGTTGGATGAAAGAGGAGGCAACAAGGAG |
|  |  |  | Extend | CCACCAAGGAGCAAGTCCCAGG |
| 7 | rs9350458 | I | Forward | ACGTTGGATGTCATTCCAACATGAAGAACC |
|  |  |  | Reverse | ACGTTGGATGAGAGCTAAAGACATGCCCAG |
|  |  |  | Extend | ACCACTGGACATACCTT |
| 8 | rs17406935 | G | Forward | ACGTTGGATGGTTTCAATCAACATCATAAGG |
|  |  |  | Reverse | ACGTTGGATGTGTGAGCAGATATGTATCCC |
|  |  |  | Extend | GGTAAACATCATAAGGTGCACA |
| 9 | rs1188974 | G | Forward | ACGTTGGATGACAGAGCGAGACTCTGTCTA |
|  |  |  | Reverse | ACGTTGGATGGTTTATTTACGATGAGGATTC |
|  |  |  | Extend | TTTACGATGAGGATTCAGATTTA |
| 10 | rs660641 | G | Forward | ACGTTGGATGATCTCACAAATGCCATGTGC |
|  |  |  | Reverse | ACGTTGGATGTCAACTAGGTGCCATATTTG |
|  |  |  | Extend | GGTCGGTGCCATATTTGGAGGCAG |
| 11 | rs11141939 | I | Forward | ACGTTGGATGCAAAGAAGGAGGGTAACTGG |
|  |  |  | Reverse | ACGTTGGATGTCATGTCGGGCATTGTTCTC |
|  |  |  | Extend | CACGGGGCTAGGGTCAGAGGATG |
| 12 | rs2296466 | G | Forward | ACGTTGGATGAACAATACAGGGTCTCTTGC |
|  |  |  | Reverse | ACGTTGGATGTTGTGACGTAGAACTGCTCC |
|  |  |  | Extend | AATCTTGGCAAGAGGCAGC |
| 13 | rs2846071 | G | Forward | ACGTTGGATGTCCCAGTCTGGTGGACAAAC |
|  |  |  | Reverse | ACGTTGGATGAGCAATGTGGAAGTCACTGG |
|  |  |  | Extend | GTGTAAGTCACTGGGGGTAATATGGAC |
| 14 | rs72983568 | I | Forward | ACGTTGGATGTGGGTGACAGAACAAGACTC |
|  |  |  | Reverse | ACGTTGGATGGCTACTTTATCTCTGGCTTC |
|  |  |  | Extend | CGGCTTCATGCCTTCA |
| 15 | rs4149577 | G | Forward | ACGTTGGATGTGTCAGTTCATCTTGCGAGG |
|  |  |  | Reverse | ACGTTGGATGTGCAATAACCCAGGTGTGAG |
|  |  |  | Extend | GAATGCGGAGAGGAC |
| 16 | rs10771523 | I | Forward | ACGTTGGATGTTCCCTCCTGTCAGATAACC |
|  |  |  | Reverse | ACGTTGGATGTTCTTGAAAGGTGTGGGTGG |
|  |  |  | Extend | CCGGGTTGGAGATCAGTTGGC |
| 17 | rs1551465 | G | Forward | ACGTTGGATGATTTACTTGGCACTGGCTCC |
|  |  |  | Reverse | ACGTTGGATGGGACATTGATCAGGAAACCG |
|  |  |  | Extend | ATTTATCAGGAAACCGGGGTCAGGA |
| 18 | rs3851793 | G | Forward | ACGTTGGATGTGAATTCTCTCACACCAGCC |
|  |  |  | Reverse | ACGTTGGATGCAATTAAACAGTCAGTGCCG |
|  |  |  | Extend | GGTTCCGCTGTTTCCTCATGTCAA |
| 19 | rs7224395 | I | Forward | ACGTTGGATGCTCTCTATTCCTAATACTTC |
|  |  |  | Reverse | ACGTTGGATGGGCTTGTGTCTTCAAAACCC |
|  |  |  | Extend | TCTCTCACTTCTTTCACC |
| 20 | rs148091530 | I | Forward | ACGTTGGATGAAATTAGCTGGGTGTGGTGG |
|  |  |  | Reverse | ACGTTGGATGACCCGGGTACAAGCGATTTT |
|  |  |  | Extend | AGCCTCCCTAGTAGC |
| 21 | rs79246064 | G | Forward | ACGTTGGATGCAAGCAGGACCAAATGTCAG |
|  |  |  | Reverse | ACGTTGGATGACCCTACTATTACTGCCTGC |
|  |  |  | Extend | AAACAAATGCCTCTTTTCTCAATCA |
| 22 | rs4402823 | G | Forward | ACGTTGGATGAGCCGACTTATCAAGACAGG |
|  |  |  | Reverse | ACGTTGGATGAATAAAGCTCCAGTCTCCCG |
|  |  |  | Extend | AACCGCATGGGTTACTTTCTCC |

SNP, single nucleotide polymorphism. Primers were used for using Sequenom MassArray System. PCR was performed with an initial 2 minutes (min) at 50°C and 10 min at 95°C, followed by 40 cycles of 15 seconds at 95°C and 1 min at 60°C. In the replication stage, 22 index SNPs were selected for genotyping. Finally, among these 22 SNPs, rs148091530 was failed to be genotyped and two SNPs (rs4149577and rs2846071) survived in the replication stage. ^a^Types of SNPs: “G” stands for the genotyped SNPs, and “I” stands for the imputed SNPs.

**Supplementary Table 6 Summary of the association results in the replication stage.**

| No. | Chr. | SNPs | Positions^a^ | Alleles^b^ | A1 hom / Het / A2 hom^c^ | | | Genotyped or  imputed^d^ | Genes^e^ | ORs (95% CIs)^f^ | *P*^g^ | Meta-analyses results^h^ | | | |
| --- | --- | --- | --- | --- | --- | --- | --- | --- | --- | --- | --- | --- | --- | --- | --- |
|  |  |  |  |  | Cases-controls | Cases | Controls |  |  |  |  | *P* | OR (95% CIs) | *P*heterogeneity | I2 |
| 1 | 2 | rs2540334 | 202271347 | T/C | 21/147/237 | 3/11/18 | 18/136/219 | G | *TRAK2* | 1.21 (0.67-2.17) | 0.522 | 1.52E-04 | 1.93 (1.37-2.72) | 0.054 | 73.05 |
| 2 | 2 | rs13030006 | 206666066 | T/C | 56/192/154 | 6/16/12 | 50/176/142 | G | *NRP2* | 1.17 (0.70-1.95) | 0.547 | 4.33E-04 | 1.81 (1.30-2.52) | 0.028 | 79.28 |
| 3 | 4 | rs6812414 | 38322807 | A/C | 3/87/314 | 1/8/25 | 2/79/289 | G | *RP11-600L4.1* | 1.39 (0.67-2.91) | 0.376 | 2.11E-04 | 2.23 (1.46-3.41) | 0.13 | 57.34 |
| 4 | 4 | rs4692983 | 94392039 | G/T | 44/109/248 | 1/7/25 | 43/102/223 | I | *GRID2* | 0.53 (0.28-1.03) | 0.0620 | 3.24E-02 | 1.53 (1.04-2.25) | 1.00E-04 | 93.25 |
| 5 | 4 | rs11936923 | 161091459 | A/T | 12/79/314 | 2/4/28 | 10/75/286 | I | *RP11-502M1.2* | 0.92 (0.44-1.90) | 0.816 | 1.43E-03 | 1.96 (1.30-2.97) | 0.013 | 83.91 |
| 6 | 5 | rs34607327 | 54001948 | T/A | 2/66/335 | 0/5/29 | 2/61/306 | I | *AC112198.2* | 0.82 (0.31-2.13) | 0.679 | 3.20E-03 | 2.42 (1.35-4.37) | 0.0049 | 87.38 |
| 7 | 6 | rs9350458 | 73133122 | A/T | 54/195/154 | 5/14/15 | 49/181/139 | I | *RIMS1* | 0.89 (0.53-1.52) | 0.678 | 3.18E-03 | 1.69 (1.19-2.39) | 0.0017 | 89.8 |
| 8 | 7 | rs17406935 | 78664922 | A/G | 2/50/352 | 0/5/27 | 2/45/325 | G | *MAGI2* | 1.20 (0.46-3.12) | 0.708 | 1.32E-04 | 2.93 (1.69-5.08) | 0.025 | 80.13 |
| 9 | 7 | rs1188974 | 158665748 | A/G | 4/65/337 | 1/3/30 | 3/62/307 | G | *WDR60* | 0.79 (0.31-2.02) | 0.627 | 2.31E-03 | 2.34 (1.35-4.04) | 0.0052 | 87.17 |
| 10 | 8 | rs660641 | 2873709 | C/T | 22/127/259 | 2/10/20 | 20/117/239 | G | *CSMD1* | 1.06 (0.58-1.93) | 0.855 | 1.09E-03 | 1.86 (1.28-2.71) | 0.018 | 82.12 |
| 11 | 9 | rs11141939 | 90290400 | G/T | 34/165/197 | 0/17/16 | 34/148/181 | I | *DAPK1* | 0.82 (0.46-1.46) | 0.495 | 5.70E-03 | 1.63 (1.15-2.30) | 0.0034 | 88.31 |
| 12 | 10 | rs2296466 | 23393091 | G/A | 8/106/286 | 1/8/25 | 7/98/261 | G | *MSRB2* | 0.95 (0.47-1.94) | 0.895 | 1.73E-03 | 1.90 (1.27-2.84) | 0.021 | 81.32 |
| 13 | 11 | rs2846071 | 75980710 | T/C | 61/173/168 | 11/12/11 | 50/161/157 | G | *WNT11* | 1.75 (1.08-2.84) | 0.0240 | 4.89E-06 | 2.14 (1.96-3.46) | 0.27 | 19.07 |
| 14 | 11 | rs72983568 | 102773998 | G/T | 3/37/362 | 0/1/33 | 3/36/329 | I | *U7* | 0.26 (0.04-1.91) | 0.187 | 1.94E-04 | 3.80 (1.88-7.67) | 0.0047 | 87.52 |
| 15 | 12 | rs4149577 | 6447522 | A/G | 51/194/165 | 8/16/10 | 43/178/155 | G | *TNFRSF1A* | 1.67 (1.00-2.78) | 0.0490 | 6.88E-06 | 2.05 (1.89-2.51) | 0.31 | 1.28 |
| 16 | 12 | rs10771523 | 9472268 | A/G | 4/70/304 | 2/5/24 | 2/65/280 | I | *SNORA75* | 1.54 (0.73-3.27) | 0.260 | 1.70E-06 | 3.20 (1.99-5.15) | 0.42 | 0 |
| 17 | 15 | rs1551465 | 101527412 | G/A | 76/221/108 | 3/18/13 | 73/203/95 | G | *LRRK1* | 0.58 (0.34-1.01) | 0.0520 | 1.64E-05 | 0.47 (0.33-0.66) | 0.33 | 0 |
| 18 | 17 | rs3851793 | 45059900 | T/C | 28/153/223 | 1/12/21 | 27/141/202 | G | *GOSR2* | 0.73 (0.40-1.34) | 0.304 | 1.39E-02 | 1.61 (1.10-2.35) | 1.00E-03 | 90.73 |
| 19 | 17 | rs7224395 | 67653207 | T/C | 61/190/153 | 6/10/17 | 55/180/136 | I | *AC003051.1* | 0.78 (0.46-1.33) | 0.361 | 8.53E-03 | 1.60 (1.13-2.27) | 4.00E-04 | 91.93 |
| 20 | 18 | rs148091530 | 12419210 | G/A | 0/0/403 | 0/0/34 | 0/0/369 | I | *SLMO1* | NA | NA | NA | NA | NA | NA |
| 21 | 19 | rs79246064 | 13317457 | C/T | 1/36/368 | 0/1/31 | 1/35/337 | G | *CACNA1A* | 0.31 (0.040-2.26) | 0.245 | 5.67E-04 | 3.39 (1.69-6.78) | 0.012 | 84.13 |
| 22 | 20 | rs4402823 | 47089525 | T/C | 76/209/122 | 6/10/15 | 70/199/107 | G | *RP5-991B18.1* | 0.66 (0.38-1.14) | 0.138 | 2.73E-05 | 0.48 (0.34-0.67) | 0.14 | 53.47 |

CI, confidence interval; Chr., chromosome; OR, odds ratio; SNP, single-nucleotide polymorphism. ^a^Positions based on GRCh37; ^b^Minor allele/major allele; ^c^Counts of Minor allele homozygote/heterozygote/major allele homozygote; ^d^Types of SNPs: “G” stands for the genotyped SNPs, and “I” stands for the imputed SNPs; ^e^Genes or the nearest genes; ^f^ORs and 95% CIs were calculated for the minor allele in the replication stage. ^g^*P* was calculated for the minor allele in the replication stage. ^h^The meta-analyses results of the discovery and replication stages. The rs148091530 was not successfully genotyped in this study.

**Supplementary Table 7 Stratification analyses of rs2846071 and rs4149577 by age.**

**(a) Stratification analysis of rs2846071 by age.**

| Categories | Genotypes | rs2846071 | | | | | |
| --- | --- | --- | --- | --- | --- | --- | --- |
|  |  | Discovery stage, n | | Replication stage, n | | Overall, n | |
|  |  | Case | Control | Case | Control | Case | Control |
| Age, years | |  |  |  |  |  |  |
| ≤ 23 | CC | 11 | 47 | 5 | 43 | 16 | 90 |
|  | TC | 13 | 53 | 11 | 65 | 24 | 118 |
|  | TT | 4 | 10 | 1 | 18 | 5 | 28 |
| ORs (95% CIs) | | 3.44 (1.66 - 7.13) | | 1.14 (0.36 - 3.63) | | 2.11 (1.20 - 3.71) | |
| *P* | | 9.1E-4 | | 0.82 | | 9.3E-3 | |
| > 23 | CC | 12 | 55 | 9 | 111 | 21 | 166 |
|  | TC | 22 | 55 | 4 | 93 | 26 | 148 |
|  | TT | 3 | 13 | 2 | 40 | 5 | 53 |
| ORs (95% CIs) | | 2.19 (1.23 - 3.91) | | 1.98 (1.17 - 3.37) | | 1.94 (1.34 - 2.80) | |
| *P* | | 7.8E-3 | | 0.010 | | 4.3E-4 | |
| *P*_heterogeneity_ | | 0.34 | | 0.40 | | 0.80 | |

**(b) Stratification analysis of rs4149577 by age.**

| Categories | Genotypes | rs4149577 | | | | | |
| --- | --- | --- | --- | --- | --- | --- | --- |
|  |  | Discovery stage, n | | Replication stage, n | | Overall, n | |
|  |  | Case | Control | Case | Control | Case | Control |
| Age, years | |  |  |  |  |  |  |
| ≤ 23 | AA | 3 | 12 | 2 | 22 | 5 | 34 |
|  | AG | 13 | 48 | 11 | 55 | 24 | 103 |
|  | GG | 12 | 50 | 6 | 52 | 18 | 102 |
| ORs (95% CIs) | | 3.59 (1.77 - 7.30) | | 1.58 (0.55 - 4.51) | | 2.36 (1.38 - 4.01) | |
| *P* | | 4.1E-4 | | 0.39 | | 0.0016E-3 | |
| > 23 | AA | 6 | 24 | 0 | 27 | 6 | 51 |
|  | AG | 16 | 47 | 9 | 119 | 25 | 166 |
|  | GG | 15 | 52 | 6 | 101 | 21 | 153 |
| ORs (95% CIs) | | 1.84 (1.13 - 2.99) | | 1.8 (0.99 - 3.30) | | 1.9 (1.30 - 2.77) | |
| *P* | | 0.015E-2 | | 0.060 | | 8.7E-4 | |
| *P*_heterogeneity_ | | 0.13 | | 0.83 | | 0.52 | |

CI, confidence interval; NA not available; OR, odds ratio. The *P* values, ORs and 95% CIs were calculated under an additive model by logistic regression adjusting for age. PLINK (v 1.90) software was used to calculate the heterogeneity of the population in the discovery and the replication stages. *P*_heterogeneity_ value of less than 0.05 was considered to be statistically significant. A total of 298 samples were included in the discovery stage in this study, and the mean age of these samples was 23 years old. Previous studies used the mean age for stratification analysis (Nat Genet. 2010;42(9):755-8; Clin Cancer Res. 2018;24(4):906-915). Therefore, we used 23 years old for stratification analysis.

**Supplementary Table 8 The predicted functional relevance of rs2846071, rs4149577 and the other SNPs in strong or moderate LD with them.**

**(a) The predicted functional relevance of rs2846071 and 8 SNPs in strong or moderate LD with rs2846071.**

| Chr. | Positions^a^ | LD (r^2^) | SNPs | Ref/Alt | *P* values in GWAS | Posterior probabilities | Promoter in brain tissues | Enhancer in brain tissues | DNAse in brain tissues | Genes^b^ |
| --- | --- | --- | --- | --- | --- | --- | --- | --- | --- | --- |
| 11 | 75965407 | 0.42 | rs643965 | T/C | 0.68 | 0.0034 | Yes | No | No | *WNT11* |
| 11 | 75966144 | 0.42 | rs678108 | T/C | 0.082 | NA | Yes | No | No | *WNT11* |
| 11 | 75966746 | 0.42 | rs2618076 | C/T | 0.13 | 0.00069 | No | No | No | *WNT11* |
| 11 | 75967072 | 0.42 | rs2851167 | T/G | 0.13 | 0.0044 | No | No | No | *WNT11* |
| 11 | 75967182 | 0.42 | rs2618077 | G/C | 0.13 | NA | No | No | No | *WNT11* |
| 11 | 75968200 | 0.43 | rs11606029 | T/C | 0.35 | NA | No | Yes | Yes | *WNT11* |
| 11 | 75980710 | 1 | rs2846071 | T/C | 3.75E-05 | 0.48 | No | Yes | No | *WNT11* |
| 11 | 75980855 | 0.4 | rs2618079 | A/G | 0.05 | 0.040 | No | Yes | No | *WNT11* |
| **(b) The predicted functional relevance of rs4149577 and 8 SNPs in strong or moderate LD with rs4149577.** | | | | | | | | | | |
| Chr. | Positions^a^ | LD (r^2^) | SNPs | Ref/Alt | *P* values in GWAS | Posterior probabilities | Promoter in brain tissues | Enhancer in brain tissues | DNAse in brain tissues | Genes^b^ |
| 12 | 6441348 | 0.5 | rs4149587 | C/G | 0.03 | NA | No | Yes | No | *TNFRSF1A* |
| 12 | 6442346 | 0.6 | rs1800692 | A/G | 3.39E-04 | 1.00 | Yes | Yes | No | *TNFRSF1A* |
| 12 | 6445329 | 0.91 | rs2284344 | G/C | 9.25E-05 | NA | Yes | Yes | No | *TNFRSF1A* |
| 12 | 6445982 | 0.99 | rs887477 | A/C | 7.89E-05 | 0.0010 | Yes | Yes | Yes | *TNFRSF1A* |
| 12 | 6447437 | 0.47 | rs4149578 | C/T | 2.74E-04 | 0.000033 | Yes | Yes | No | *TNFRSF1A* |
| 12 | 6447522 | 1 | rs4149577 | G/A | 3.09E-05 | 0.0021 | Yes | Yes | No | *TNFRSF1A* |
| 12 | 6451590 | 0.57 | rs4149570 | A/C | 4.14E-04 | 1.00 | Yes | Yes | No | *TNFRSF1A* |
| 12 | 6455098 | 0.5 | rs11064145 | T/G | 0.13 | 4.02E-07 | Yes | Yes | No | *TNFRSF1A* |

Chr., chromosome; LD, linkage disequilibrium; NA, not available; SNP, single nucleotide polymorphism. Posterior probabilities were calculated by Probabilistic Annotation INTegratOR (PAINTOR), *r*^2^ was calculated between the rs2846071 or rs4149577 and the other SNPs based on genotype data of the Asian population from the 1,000 Genomes Project (phase 3). Only those SNPs with *r*^2^ > 0.4 were retained. ^a^Positions based on NCBI Build 37; ^b^Genes or nearest genes. These data were calculated using HaploReg (v4.1).**Supplementary Table 9 Pathway analyses based on i-GSEA4GWAS.**

| Pathways | *P* values | FDR | Significant genes |
| --- | --- | --- | --- |
| Arachidonic acid metabolism | < 0.0010 | 4.62E-04 | *CYP4F3, CYP2C9, CYP2C19, CYP2C18, CYP2C8, PLA2G2D, ALOX15B, LTA4H, PLA2G1B, PTGES2, EPHX2,  PLA2G2A, PTGES, CYP2E1, ALOX15, TBXAS1, CYP4A11, CYP2J2, LTC4S, AKR1C3* |
| Inositol phosphate metabolism | < 0.0010 | 7.68E-04 | *PLCG2, INPP5A, PLCD4, PIP5K1C, PLCB1, PTEN, PLCB4, PIK3CB, PIP5K1B, MINPP1, SYNJ2, ITPKB, PLCG1,  INPP4B, PLCB3, PLCZ1, ITPK1, PLCE1, PIK3C3, SYNJ1* |
| Notch signaling pathway | < 0.0010 | 1.26E-03 | *NOTCH3, CTBP2, NOTCH2, NCOR2, MAML2, MAML3, DTX1, SNW1, MFNG, DTX2, CREBBP, HES1, NCSTN, DVL2,  JAG1, NUMB* |
| Wnt signaling pathway | 0.0020 | 4.99E-03 | *MAPK10, PRKCI, PRKCH, PPP2R5E, PRKCQ, FRAT1, PRKCE, WNT3, AXIN1, WNT2B, WNT11, CCND3, CCND1, PRKCA, FZD10, WNT5B, CCND2, PAFAH1B1, PRKD1, DVL2, LDLR, SFRP4, WNT7A, WNT2, FZD8, FZD5, WNT16, RAC1* |
| Tumor necrosis factor pathway | 0.044 | 8.17E-02 | *TNFRSF1A, CASP8, CFLAR, TNFRSF1B, CASP3, MAP3K7, RALBP1, NFKBIA* |

A total of 3,819,674 single nucleotide polymorphisms (SNPs) were input into the i-GSEA4GWAS software. To avoid overrepresentation of SNPs in more than one gene, we restricted mapping SNPs to +/-20 kilobases (kb) around a gene. The canonical pathway of gene-sets was used for further analysis, which was extracted and curated from the Molecular Signatures Database (MSigDB). The false discovery rate (FDR) of less than 0.1 was considered to be statistically significant.

**Supplementary Table 10 The allele and genotype frequencies of rs2846071 and rs4149577 in different populations.**

| **(a) The allele and genotype frequencies of rs2846071 in different populations.** | | | | | | | | |
| --- | --- | --- | --- | --- | --- | --- | --- | --- |
| Populations | Sample size^a^, n | Frequencies of T allele | Alleles, n | | Genotypes, n | | | *P* |
|  |  |  | T | C | TT | TC | CC |  |
| In the present study |  |  |  |  |  |  |  |  |
| Discovery stage samples | 298 | 0.341 | 203 | 393 | 30 | 143 | 125 |  |
| Replication stage samples | 404 | 0.367 | 295 | 509 | 61 | 173 | 168 |  |
| Overall | 702 | 0.356 | 498 | 902 | 91 | 316 | 293 |  |
| From the 1,000 Genomes Project^b^ |  |  |  |  |  |  |  |  |
| CHB and CHS | 208 | 0.358 | 149 | 267 | 21 | 107 | 80 | 0.93 |
| African descent | 661 | 0.780 | 1037 | 293 | 403 | 223 | 35 | 2.39E-110 |
| American descent | 347 | 0.537 | 373 | 321 | 103 | 167 | 77 | 1.97E-15 |
| East Asian descent | 504 | 0.354 | 357 | 651 | 57 | 243 | 204 | 0.94 |
| European descent | 503 | 0.674 | 678 | 328 | 230 | 218 | 55 | 1.56E-53 |

**(b) The allele and genotype frequencies of rs4149577 in different populations.**

| Populations | Sample size^a^, n | Frequencies of A allele | Alleles, n | | Genotypes, n | | | *P* |
| --- | --- | --- | --- | --- | --- | --- | --- | --- |
|  |  |  | A | G | AA | AG | GG |  |
| In the present study |  |  |  |  |  |  |  |  |
| Discovery stage samples | 298 | 0.359 | 214 | 382 | 45 | 124 | 129 |  |
| Replication stage samples | 410 | 0.361 | 296 | 524 | 51 | 194 | 165 |  |
| Overall | 708 | 0.360 | 510 | 906 | 96 | 318 | 294 |  |
| From the 1,000 Genomes Project^b^ |  |  |  |  |  |  |  |  |
| CHB and CHS | 208 | 0.370 | 154 | 262 | 27 | 100 | 81 | 0.71 |
| African descent | 661 | 0.923 | 1220 | 102 | 564 | 92 | 5 | 2.25E-204 |
| American descent | 347 | 0.488 | 339 | 355 | 86 | 167 | 94 | 1.64E-08 |
| East Asian descent | 504 | 0.366 | 369 | 639 | 69 | 231 | 204 | 0.77 |
| European descent | 503 | 0.528 | 531 | 475 | 139 | 253 | 111 | 2.15E-16 |

The sample size was based on the number of successfully genotyped individuals. ^b^The 1,000 Genomes Project data was based on the release in November 2014 (phase 3). *P* value is the result of the chi-square test, and a *P* value less than 0.05 is considered to be statistically significant. Populations of African descent include the Americans of African Ancestry in SW USA (ASW), African Caribbeans in Barbados (ACB), Esan in Nigeria (ESN), Gambian in Western Divisions in the Gambia (GWD), Luhya in Webuye, Kenya (LWK), Mende in Sierra Leone (MSL), and Yoruba in Ibadan, Nigeria (YRI). Populations of American descent include the Colombians from Medellin, Colombia (CLM), Mexican Ancestry from Los Angeles USA (MXL), Peruvians from Lima, Peru (PEL), and Puerto Ricans from Puerto Rico (PUR). Populations of East Asian descent include Chinese Dai in Xishuangbanna, China (CDX), Han Chinese in Beijing, China (CHB), Han Chinese South (CHS), Japanese in Toyko, Japan (JPT), and Kinh in Ho Chi Minh City, Vietnam (KHV). Populations of European descent include the British in England and Scotland (GBR), Utah Residents (CEPH) with Northern and Western European ancestry (CEU), Finnish in Finland (FIN), Iberian Population in Spain (IBS), and Toscani in Italia (TSI).

**References**

1. Ward LD, Kellis M: **HaploReg v4: systematic mining of putative causal variants, cell types, regulators and target genes for human complex traits and disease**. *Nucleic Acids Res* 2016, **44**(D1):D877-881.

2. Gong Y, Greenbaum J, Deng HW: **A statistical approach to fine-mapping for the identification of potential causal variants related to human intelligence**. *J Hum Genet* 2019, **64**(8):781-787.

3. Zhang K, Chang S, Guo L, Wang J: **I-GSEA4GWAS v2: a web server for functional analysis of SNPs in trait-associated pathways identified from genome-wide association study**. *Protein Cell* 2015, **6**(3):221-224.

4. Liberzon A, Subramanian A, Pinchback R, Thorvaldsdottir H, Tamayo P, Mesirov JP: **Molecular signatures database (MSigDB) 3.0**. *Bioinformatics* 2011, **27**(12):1739-1740.

5. Wang J, Heng YJ, Eliassen AH, Tamimi RM, Hazra A, Carey VJ, Ambrosone CB, de Andrade VP, Brufsky A, Couch FJ *et al*: **Alcohol consumption and breast tumor gene expression**. *Breast Cancer Res* 2017, **19**(1):108.
